# Phenol and Polyaromatic Hydrocarbons Are Stronger Drivers Than Host Plant Species in Shaping the Arbuscular Mycorrhizal Fungal Component of the Mycorrhizosphere

**DOI:** 10.3390/ijms232012585

**Published:** 2022-10-20

**Authors:** Monika Malicka, Franco Magurno, Zofia Piotrowska-Seget

**Affiliations:** Institute of Biology, Biotechnology and Environmental Protection, Faculty of Natural Sciences, University of Silesia in Katowice, Jagiellońska 28 Street, 40-032 Katowice, Poland

**Keywords:** arbuscular mycorrhizal fungi, LSU rDNA, metagenomics, phenol, PAHs

## Abstract

Changes in soil microbial communities in response to hydrocarbon pollution are critical indicators of disturbed ecosystem conditions. A core component of these communities that is functionally adjusted to the life-history traits of the host and environmental factors consists of arbuscular mycorrhizal fungi (AMF). AMF communities associated with *Poa trivialis* and *Phragmites australis* growing at a phenol and polynuclear aromatic hydrocarbon (PAH)-contaminated site and at an uncontaminated site were compared based on LSU rDNA sequencing. Dissimilarities in species composition and community structures indicated soil pollution as the main factor negatively affecting the AMF diversity. The AMF communities at the contaminated site were dominated by fungal generalists (*Rhizophagus*, *Funneliformis*, *Claroideoglomus*, *Paraglomus*) with wide ecological tolerance. At the control site, the AMF communities were characterized by higher taxonomic and functional diversity than those exposed to the contamination. The host plant identity was the main driver distinguishing the two AMF metacommunities. The AMF communities at the uncontaminated site were represented by *Polonospora*, *Paraglomus*, *Oehlia*, *Nanoglomus*, *Rhizoglomus*, *Dominikia*, and *Microdominikia*. *Polonosporaceae* and *Paraglomeraceae* were particularly dominant in the *Ph. australis* mycorrhizosphere. The high abundance of early diverging AMF could be due to the use of primers able to detect lineages such as *Paraglomeracae* that have not been recognized by previously used 18S rDNA primers.

## 1. Introduction

The Oil Age has undoubtedly been one of the most prominent eras of human development, but also a time of man-made environmental degradation. The dynamic expansion of our civilization has resulted in the global contamination of lands and waters with toxic organic pollutants, including petroleum hydrocarbons and their derivatives, that have a detrimental impact on the biodiversity and functioning of ecosystems. The engines of ecosystem performance are soil microorganisms. Complex microbial communities formed by rhizobacteria, endophytes and mycorrhizal fungi cooperate with plants to decrease the toxicity of contaminants by rhizodegradation or phytostabilization [1,2]. Exploration of changes in the structure of microbial communities in response to hydrocarbon pollution is critical for understanding their role in maintaining ecosystem integrity and evaluating the condition of disturbed ecosystems [3,4].

Arbuscular mycorrhizal fungi (AMF) representing the basal fungal lineage of Glomeromycota are obligated plant mutualists acting as key suppliers of phosphorus and other nutrients to the vast majority of land plants in exchange for plant-derived sugars and lipids [5]. Their co-evolution with plants has favored the selection of AMF communities functionally adjusted to the life-history traits of the host as well as to environmental conditions on a global and local scale [6,7]. Mycorrhizal colonization and AMF community structures can be significantly affected by several edaphic factors such as soil pH, texture, moisture, and the contents of macro- and micronutrients [8,9,10]. Soil pH is one of the strongest environmental drivers of AMF diversity. The core of AMF communities in acidic soils is represented by generalists that also exhibit wider tolerance to other abiotic stresses because of their phenotypically plastic genotypes [11]. It has been shown that low soil pH inhibits extraradical mycelial growth and spore formation in vulnerable AMF species [12]. On the other hand, pH increases are associated with a higher functional and taxonomic AMF diversity without detrimental effects on generalist AMF because of their low vulnerability to changes in soil pH [11]. Among other factors, soil pollution and host plant species can play a key role in shaping AMF assemblages by exerting selective pressure through toxicity and nutrient trading [13,14,15,16]. Plants can also indirectly influence AMF diversity by changing soil physicochemical properties through exudates released by a root system [8,16].

A small number of studies on the diversity of AMF communities in soils contaminated with oil hydrocarbons have demonstrated low AMF species richness and the dominance of AMF generalists of high ecological tolerance [13,17,18]. Our previous investigation has confirmed the negative effect of phenol and polynuclear aromatic hydrocarbons (PAHs) on AMF diversity and the high relative abundances of generalist taxa such as *Rhizoglomus* and *Funneliformis* in the communities [19]. A follow-up experiment revealed the high potential of *Funneliformis caledonius*, isolated from a contaminated site, to promote the growth of plants under phenol and PAH exposure [20]. The analysis of the diversity of AMF communities performed by Malicka et al. [19] was based on the sequencing of small subunit ribosomal DNA (SSU rDNA) using a denaturing gradient gel electrophoresis (DGGE) approach, which provided only preliminary data. 

In molecular studies on fungal diversity, the most popular, cost-effective, and high-resolution approach is based on Illumina’s next generation sequencing (NGS). The molecular marker routinely used to characterize the total fungal communities is the nuclear ribosomal internal transcribed spacer (ITS) [21]. Despite being a universal fungal barcode suitable for detecting ectomycorrhizal fungi, represented mainly by Ascomycota and Basidiomycota, the ITS subunit alone does not represent an efficient molecular marker for AMF. Glomeromycota are characterized by higher inter- and intraspecies genetic variability than other fungal taxa and are poorly represented in the environmental pools of ITS amplicons [22]. The SSU rDNA portion is a more suitable region commonly used in the NGS-based analysis of AMF biodiversity. This marker is reliably supported by the MaarjAM database of SSU rDNA AMF sequences [23]. Nonetheless, the SSU subregion lacks the necessary resolution power to distinguish closely related AMF species [24,25,26]. The best barcoding marker for distinguishing closely related AMF species is, up to now, the ≈ 1500 bp region covering the partial SSU rDNA, ITS and the partial LSU rDNA [27]. It is well-supported by a large number of reference sequences deposited in the dataset of [28] and GenBank (NCBI). However, due to its size, the use of this marker in commercial short-read Illumina sequencing is not possible. It requires the application of other technologies such as PacBio single-molecule real-time sequencing and Oxford Nanopore sequencing, which are costly, burdened with a high error rate and less accessible than Illumina [29,30]. A feasible alternative is represented by the LSU rDNA, which provides a better taxonomic resolution than SSU rDNA due to the presence of the D2 hypervariable region [31]. In the present work, we provide a detailed analysis of the diversity of AMF communities affected by soil contamination with toxic organic pollutants based on the NGS workflow. Given the difficulties of finding suitable LSU primers specific for Glomeromycota and amplifying a portion with a size compatible with the Illumina requirements, we tested the possibility of using a newly designed set of LSU primers which were not Glomeromycota-specific. Despite the non-specificity in the annealing step, preliminary analysis in silico proved that the size of the amplicons enabled the selection of Glomeromycota bands after an electrophoresis run on an agarose gel.

## 2. Results

### 2.1. Soil Physicochemical Features

Soil samples, characterized by granulometry as loamy sand, had similar physicochemical parameters at both sites, differing only in the concentrations of phenol and PAHs, and conductivity (Table S1). However, a higher soil conductivity at the contaminated site was due to the presence of steel filings from post-industrial wastes that are not a bioavailable form of iron. The concentrations of contaminants ranged from 98 to 2814 mg of phenol per kg soil^−1^ and from 40 to 538 mg of PAHs per kg soil^−1^ at the contaminated site. The main PAHs detected in the contaminated soil were naphthalene, phenanthrene, pyrene, fluoranthene and dibenzo(a,h)anthracene. The concentrations of phenol and PAHs at the uncontaminated site were below the detection threshold. The average soil pH_water_/pH_KCl_ at the contaminated and uncontaminated sites were 7.13/7.31 and 6.15/5.63, respectively. The average contents of soil organic matter (SOM) at the contaminated and uncontaminated site were 4.85 and 3.96%, respectively. The average soil moisture at the contaminated and uncontaminated site was 6.88 and 4.96%, respectively. The average contents of total N at the contaminated and uncontaminated sites were 0.61 and 0.68%, respectively. The detailed description of soil physicochemical features is available in Table S1 and in the previous report of Malicka et al. [19].

### 2.2. PCR Results

PCR products after amplification with FULF and FULR primers were separated in an agarose gel giving 3–4 bands per sample: two bands < 300 bp presumably containing the amplicons from Ascomycota and Basidiomycota, and two bands in the range of 330–480 bp expected to represent the pool of Glomeromycota amplicons (Figure S1A). After extraction, the latter were used as a template for nested PCR with the primers FULFN1ngs/FULFN2ngs–FULRNngs (Figure S1B) The qualitative analysis of the indexed libraries with Agilent 2200 TapeStation System showed the presence of the expected 390–510 bp products (Figure S2).

### 2.3. Sequences Processing 

In total, 11,175,408 contigs were assembled as the result of the sequencing of 96 libraries. A total of 8,086,118 sequences were removed after the filtering step for quality and size (<300 bp and >470 bp). After a classification and chimera search, the remaining 3,089,290 sequences were thinned to 1,147,109 sequences. The sequences were clustered in 686 taxonomic units that were further assigned to 90 species using RAxML-EPA and GAPPA. Nineteen of these assignments were excluded from further analysis because they were represented by less than ten sequences, or less than 100 sequences when not assigned at species level, or included pseudogenes (File S1). Ten samples representing AMF communities associated with *Phragmites australis* at the contaminated site (six root samples and four soil samples) and three samples representing AMF communities associated with *Poa trivialis* from the uncontaminated site (two root samples and one soil sample) were excluded from the further analysis because of the low number of sequences (<100). Rarefaction curves (Figure S3) and Good’s coverage >96% showed the high quality and depth of sequencing of the analyzed samples.

### 2.4. α-Biodiversity of AMF Communities

Soil contamination was identified as the main factor that affected the α-biodiversity of AMF communities. The values of observed/estimated species richness and Shannon diversity index were approx. two times higher for the AMF communities at the uncontaminated site (8–20 species) compared with the AMF communities associated with the contaminated site (4–10 species) (Table 1). The differences in the values of α-biodiversity estimators between the sites were more significant for the AMF communities associated with *P. trivialis* compared with *Ph. australis*. No significant differences were observed at either sampling site between the AMF communities associated with the two host plant species. The numbers of AMF species reported in roots were higher than in soil, but these differences were not significant. There were no differences in species evenness between the studied communities.

### 2.5. β-Biodiversity of the AMF Communities

**Figure 1:**
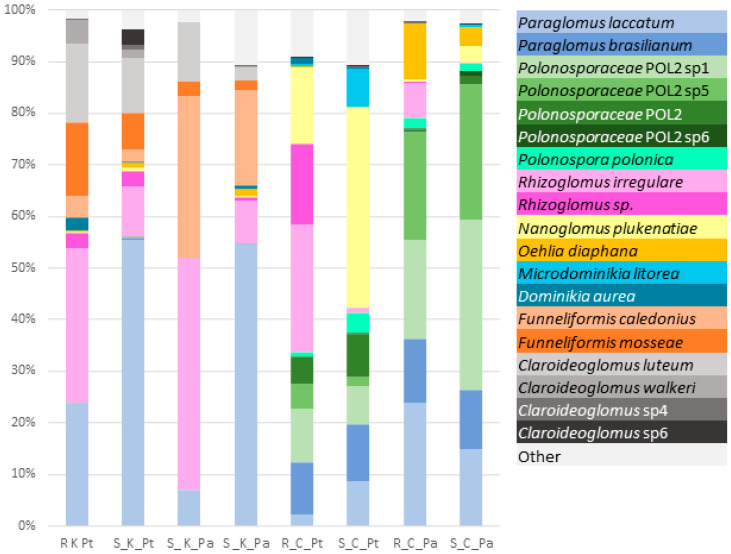
The percentage of relative species abundance in the root (R) and soil (S) AMF communities associated with *P. trivialis* (Pt) and *Ph. australis* (Pa) at the contaminated (K) and uncontaminated site (C).

**Figure 2:**
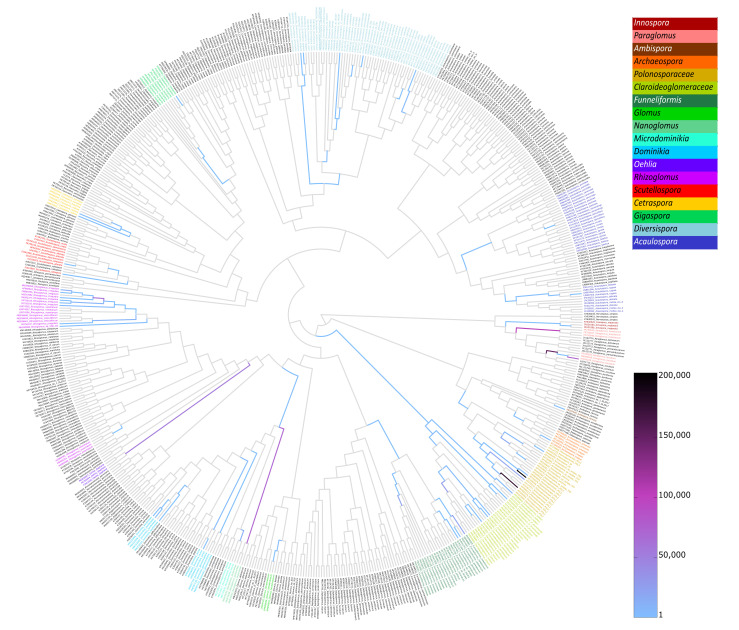
A summary heat-tree with edges colored according to the sequence placement masses of all AMF communities.

The relative species abundances (Figure 1) and placement masses (Figure 2 and Figure S4) of the AMF species revealed that the compositions of the AMF communities associated with the contaminated site and the control site were markedly different. The AMF communities at the uncontaminated site had different species compositions depending on the plant species. In contrast, the compositions of AMF communities at the contaminated site were similar for *P. trivialis* and *Ph. australis*. The dominant AMF species at the contaminated site were *Paraglomus laccatum* and *Rhizoglomus irregulare*. The relative abundance of *P. laccatum* was the highest in soil AMF communities (~55%), whereas the abundance of *R. irregulare* was the highest in roots (~40%). Other AMF species with high relative abundance at the contaminated site were *F. caledonius*, *Funneliformis mosseae*, *Claroideoglomus luteum* and *Claroideoglomus walkeri*. AMF communities associated with *Ph. australis* at the uncontaminated site were dominated by two phylotaxa of *Polonosporaceae* (~25% each species), *P. laccatum* (~20%) and *Paraglomus brasilianum* (>10%). Other AMF species abundant in these communities were *Oehlia diaphana*, *Nanoglomus plukenatiae* and *R. irregulare* (in roots). *Polonosporaceae* sp., *P. laccatum* and *P. brasilianum* were also substantial components of the AMF communities associated with *P. trivialis* at the uncontaminated site; however, these communities were dominated by *N. plukenatiae* in soil (~40%) and *R. irregulare* and *Rhizoglomus* sp. in roots (~40%). 

**Figure 3:**
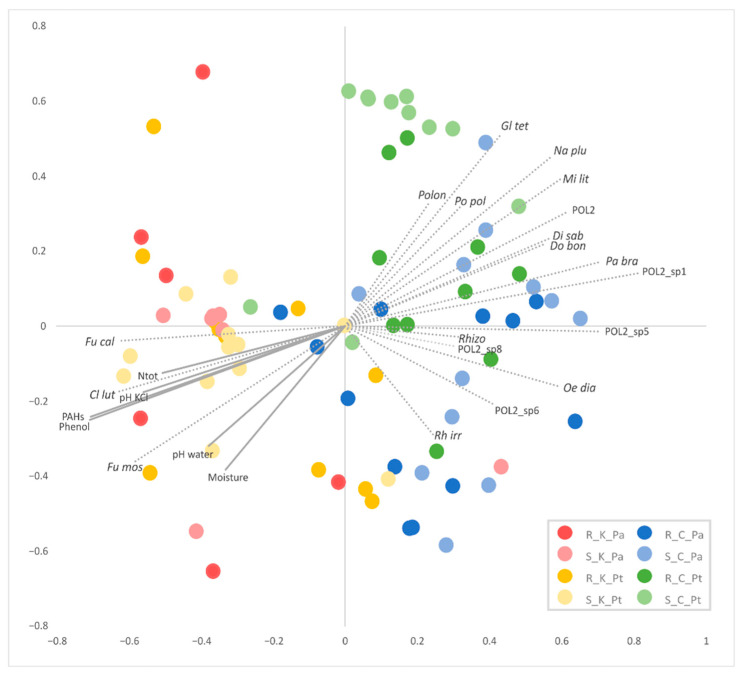
NMDS ordination based on the Yue and Clayton dissimilarity measure between the soil (S) and root (R) AMF communities associated with *P. trivialis* (Pt) and *Ph. australis* (Pa) collected from the contaminated (K) and uncontaminated (C) sites. Plot vectors indicate strength and direction of significant correlation (*p* < 0.05) between the concentrations of phenol and PAHs in soil, soil physicochemical features and the most representative AMF species. Species abbreviations: Cl lut—*Claroideoglomus luteum*, Di sab—*Diversispora sabulosa*, Do bon—*Dominikia bonfanteae*, Fu cal—*Funneliformis caledonius*, Fu mos—*Funneliformis mosseae*, Gl tet—*Glomus tetrastratosum*, Mi lit—*Microdominikia litorea*, Na plu—*Nanoglomus plukenatiae*, Oe dia—*Oehlia diaphana*, Pa bra—*Paraglomus brasilianum*, POL2—*Polonosporaceae* POL2, Polon—*Polonospora* sp., Po pol—*Polonospora polonica*, Rh irr—*Rhizoglomus irregulare*, Rhizo—*Rhizoglomus* sp.

**Figure 4:**
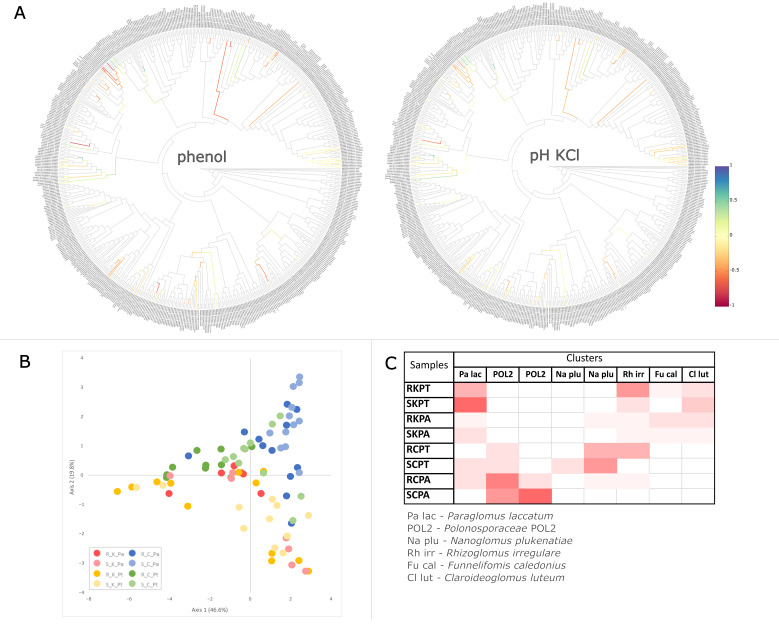
Exemplary trees visualizing Spearman’s correlation coefficient between phenol contamination and soil pH_KCl_ and edge masses. Blue and red colors indicate positive and negative correlation of placements with the phenol and pH_KCl_, respectively (**A**). Correlation trees for the other physicochemical factors (PAHs, soil moisture, SOM, N_tot_, pH_water_) are shown in Figure S7. Edge PCA demonstrating the spatial distribution of the soil (S) and root (R) AMF communities associated with *P. trivialis* (Pt) and *Ph. australis* (Pa) collected from the contaminated (K) and uncontaminated (C) sites based on the differences in placements across the edges of the phylogenetic tree (**B**). A heat-map summarizing the results of imbalance k-means clustering. Color intensity is proportional to the number of samples representing the root (R) and soil (S) AMF communities associated with *P. trivialis* (PT) and *Ph. australis* (PA) at the contaminated (K) and the uncontaminated site (C) that are assigned to clusters represented by the AMF species of the highest Euclidean distance of the edge imbalance between samples (**C**).

**Figure 5:**
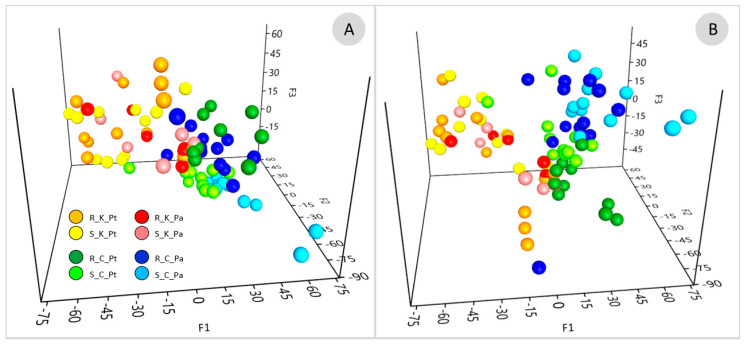
The ordination based on placement-factorization showing the distribution of root (R) and soil (S) AMF communities associated with *P. trivialis* (Pt) and *Ph. australis* (Pa) at the contaminated (K) and the uncontaminated site (C). Placement-factorization indicates the relationship between changes in the abundance of clades in the phylogenetic tree and changes is soil-physicochemical features assessed by the generalized linear model (GLM). Axes represent the balances of the winning edges of the first three phylofactors (F1, F2 and F3, Figure S5). Placement-factorization was performed considering only phenol and PAH contamination (**A**) and with all meta-data (phenol and PAH contamination, Ntot, SOM, pH_KCl_ pH H_2_O, moisture) (**B**).

Non-metric dimensional scaling (NMDS) based on the species proportions (Figure 3) and the edge principal component analysis (PCA) based on phylogenetic dissimilarity (Figure 4B) divided most of the AMF communities associated with the contaminated and uncontaminated sites into separate clusters. The significance of sample separation by NMDS was supported by the non-parametric analysis of molecular variance (AMOVA) (Table 2). The comparison of the AMF communities based on the correlation of species abundance with NMDS axes (Figure 3), the Metastats method (Table S2), placement-factorization (Figure 5, Figures S5 and S6) and edge correlation (Figure 4A and Figure S7) revealed the AMF species with the highest abundance/edge mass dissimilarity between the communities and the most important factors determining this dissimilarity. The strongest positive correlations between the relative species abundance/placement masses on the phylogenetic tree and phenol and PAH contamination were demonstrated for *C. luteum*, *F. caledonius* and *F. mosseae*. On the other hand, the strongest negative correlation with the concentrations of contaminants was found for the taxa belonging to *Polonosporaceae*, *Glomus tetrastratosum*, *Microdominikia litorea*, *Dominikia bonfanteae*, *Diversispora sabulosa* and *P. brasilianum* (Figure 3, Figure 4A and Figure S7). The Metastats method confirmed the significant dissimilarity in the abundance of these AMF species between the sites (Table S2). The edges of the phylogenetic tree that contributed the most to the separation of samples between the sites according to Edge PCA represented *Polonosporaceae*, *P. laccatum* and *Rhizoglomus* sp. (Figure S8). 

Although no significant dissimilarities in the AMF species abundance were found between the communities associated with *P. trivialis* and *Ph. australis* at the contaminated site, some significant associations between the AMF species abundance and plant species were shown at the uncontaminated site (Table S2). Placement-factorization (Figure 5, Figures S5 and S6) revealed that the dissimilarity of the AMF communities between plant species at the control site was influenced by soil pH. The phylofactors separating the AMF communities associated with different plants according to soil pH_KCl_ were determined by the differences in placement masses of *Polonosporaceae*_POL2, *P. laccatum* and *P. brasilianum* (Figure S6). The relative abundances/placement masses of *P. laccatum*, *Polonosporaceae* sp. and *O. diaphana* associated with *Ph. australis* at the control site were not significantly affected by the edaphic factors, whereas they were negatively correlated with soil moisture and pH in the case of *N. plukenatiae*, *Rhizoglomus* sp., *R. irregulare* and *M. litorea* associated with *P. trivialis* (Figure 3 and Figure S7).

A Dirichlet multinomial mixtures (DMM) model (Figure S9, Table S3) supported by imbalance k-means clustering (Figure 4C) partitioned the AMF communities into three metacommunities of significantly different species compositions. The first metacommunity was associated with the contaminated site, with no differentiation between the host plant species. The second and the third metacommunities were associated with *Ph. australis* and *P. trivialis*, respectively, at the uncontaminated site. The AMF species with the highest contribution in the first metacommunity at the contaminated site were *P. laccatum*, *R. irregulare*, *F. mosseae*, *C. luteum*, *F. caledonius*, *Rhizoglomus* sp., *F. geosporus* and *C. claroideum*. *Polonosporaceae* sp., *P. laccatum*, *P. brasilianum* and *O. diaphana* defined the second metacommunity associated with *Ph. australis* at the uncontaminated site. Partitioning of the third AMF metacommunity was determined by *N. plukenatiae*, *M. litorea*, *Glomeraceae* sp., and *Polonosporaceae* sp.

## 3. Discussion

The present study focused on the characterization of root and soil AMF communities associated with *P. trivialis* and *Ph. australis* that dominated in the plant communities growing at the shores of Kalina Pond, contaminated with phenol and PAHs, and Kokotek Pond, the uncontaminated control site. Our analysis revealed that phenol and PAH pollution was the main factor shaping the AMF communities in terms of species richness and abundance. At the control site, the host plant identity was the main driver for the differentiation of two distinct AMF metacommunities. The NGS analysis confirmed the former results obtained by DGGE profiling [19] that indicated soil contamination as the main factor affecting the species richness, abundance, and structure of the AMF communities. Except for *Paraglomeraceae* and *Polonosporaceae*, which were undetected by SSU rDNA-specific primers, AMF genera recognized as dominants by the sequencing of LSU rDNA were also reported by the previous sequencing of SSU rDNA bands from the DGGE gel. Our preliminary [19] and present studies are the first surveys on AMF diversity in a habitat exposed to several decades of phenol and PAH contamination, demonstrating its dramatic effects on AMF diversity.

The AMF communities inhabiting the contaminated soil accounted for four to ten species, which half the number at the uncontaminated site. The number of AMF operational taxonomic units (OTUs) reported in other studies conducted at sites contaminated with petroleum hydrocarbons ranged from eight to 30 OTUs [13,17,18,32,33,34]; however, in these studies the OTUs were not grouped according to their species affiliation.

The AMF communities at Kalina Pond were dominated by AMF species representing *Glomeraceae* (*R. irregulare*, *F. caledonius*, *F. mosseae*), *Claroideoglomeraceae* (*C. luteum*, *C. walkeri*) and *Paraglomeraceae* (*P. laccatum*) families. *Glomeraceae* was previously reported as the most common AMF group at hydrocarbon-contaminated sites in a temperate climate [13,18,26,27]. The relative abundance of *Rhizoglomus* sp. at high-contamination sites ranged between 65 and 98% of the total AMF communities and decreased at low-contamination sites [13,14,17,18,34]. The concentrations of hydrocarbons at Kalina Pond were lower than those reported in other studies [13,14,17,18,34], probably explaining the lower relative abundance (40%) of *R. irregulare* in comparison with other reports and the high presence of other taxa. *Claroideoglomeraceae* have been previously found at contaminated sites in AMF communities not highly dominated by *Glomeraceae* [13,14,17,18,34]. The common occurrence of widespread generalists such as *Rhizoglomus*, *Funneliformis* and *Claroideoglomus* at sites contaminated with hydrocarbons, heavy metals and mechanically disturbed areas suggests their high tolerance to abiotic stress [13,34,35,36]. Their wide ecological tolerance and dominance in stressful environments can be attributed to low host specificity and short life cycles characterized by rapid growth, extensive spore production, and biomass allocation to intraradical mycelium [37,38,39,40,41,42]. Furthermore, AMF representing *Glomeraceae* might be capable of supporting the fitness and growth of plants inhabiting these harsh environments. Malicka et al. [20], studying the effect of *F. caledonius* isolated from plants on the shore of Kalina Pond exposed to phenol and PAHs has demonstrated its beneficial effect on plant growth and mitigation of oxidative stress. 

The presence of phenol and PAHs in the contaminated soil ‘masked’ the effect of other factors on AMF diversity. Other studies also showed no effect of plant species on the diversity of AMF communities exposed to very high concentrations of hydrocarbons in Canadian soils [17,34]. Plant species, together with sample type (root/soil) and soil physicochemical features influenced the diversity of AMF communities at the control site. The AMF communities associated with *Ph. australis* had lower α-diversity compared with *P. trivialis*. *P. trivialis* and *Ph. australis* are perennial ruderal grasses with different morphology and life forms (hemicryptophyte and halophyte, respectively) that differently affect the diversity of microorganisms associated with roots, as was shown previously by Malicka et al. [19]. The divergence in functional features of these plants such as root carbon flux, N and P demand, photosynthetic efficiency, root architecture, and the composition of root exudates could strongly influence the AMF community structure [43,44]. Moreover, our analysis suggested that the differences in the diversity of AMF communities at the uncontaminated site might be also driven by edaphic soil characteristics related to the plant habitat, primarily soil moisture and pH.

Dominant species in the AMF communities at the uncontaminated site included *Polonosporaceae* sp., *P. laccatum*, *P. brasilianum*, *O. diaphana*, *N. plukenatiae* and *R. irregulare*. The relative abundances of *Funneliformis* and *R. irregulare* in these communities were remarkably lower than at the contaminated site. *R. irregulare* was abundantly present (~25% of relative abundance) only in the AMF communities in *P. trivialis* roots and accounted for a few percent of relative abundance in the other AMF communities associated with the control site. The relative abundance of *Glomeraceae* in the communities associated with *Ph. australis* was lower than in *P. trivialis* associated communities as they were dominated by representatives of *Polonosporaceae* and *Paraglomeraceae*. The presence of AMF species representing different phylogenetically-distant families of Glomeromycota at the uncontaminated site shows that these AMF communities were ecologically balanced and characterized by higher taxonomic and functional diversity than the AMF communities exposed to phenol and PAHs. A low abundance of ruderal *Glomeraceae* such as *Rhizoglomus* and *Funneliformis* that can easily dominate an environmental niche provides space for the development of other *Glomeraceae* such as *Oehlia*, *Nanoglomus*, *Dominikia* and *Microdominikia*. Some of these genera have been reported as significant components of AMF communities of contrasting habitats such as well-balanced, fertile chernozem croplands and post-mining sites where they were recognized among pioneer AMF species [45,46,47]. Possibly, the lack of contamination allows the development of AMF with a slower growth rate that allocates more resources to the formation of hyphal networks as opposed to generalist AMF proliferating in close root proximity [6].

A peculiar feature of the studied AMF communities was a very high abundance of *Paraglomeraceae* and *Polonosporaceae*. *Polonosporaceae* is a newly recognized family in the order *Archaeosporales* [48]. Before the recognition of this new family, sequences grouping within this taxon were detected in several environmental studies, proving a worldwide distribution of its members [28,49]. *Paraglomeraceae* belongs to the early divergent order *Paraglomerales*. Its ecology and the characteristics of its symbiotic associations are not well understood. Supposedly, *Paraglomeraceae* are distributed worldwide [23,50] but might remain undetected in the environment when the SSU rDNA gene is used as a barcode for AMF. AMF specific primers targeting the SSU rDNA gene have a poor match in *Paraglomeraceae* due to its high degree of divergence from other AMF lineages [51,52]. Additionally, low intraradical biomass and limited production of spores compared with other AMF might reduce the representation of *Paraglomeraceae* in the pool of environmental DNA [42,53]. Possibly, the nested-PCR approach used in this study to enrich the fraction of AMF-specific amplicons substantially improved the detection of *Paraglomeraceae* in the AMF communities. Recent studies have revealed that *Paraglomeraceae* were significant components of the AMF communities associated with uncontaminated, low hydrocarbon- or radioactive cesium-contaminated sites [13,14,54]. Similar to ruderal *Glomeraceae*, the members of *Paraglomeraceae* have a short life cycle; however, they allocate their biomass to an extensive extraradical mycelium [53,55]. Allegedly, *Paraglomeraceae* provide low benefits to their host plants compared with other, phylogenetically younger taxa such as *Glomeraceae* [50]. However, their high dominance at both sites in this study suggests that their role and occurrence could have been underestimated and biased by the choice of primers and should be investigated further. Recent genome sequencing of *Paraglomus occultum* revealed its relatively small size and fewer genes and repeats compared with most AMF. It was suggested that *Paraglomeraceae* could undergo lineage-specific adaptive genome reduction to thrive under environmental stress. Still, verification of this hypothesis requires further studies on the genomes of early divergent AMF lineages [56]. 

Besides the contamination and host plant identity, other factors that were not directly investigated in this study could affect the composition of AMF assemblages. Recent studies have revealed that changes in bacterial communities were closely related to changes in AMF communities and vice versa [14,18,19]. Some bacterial taxa such as *Actinomycetes* that are often associated with hydrocarbon-contaminated soils might be AMF antagonists, whereas others such as *Proteobacteria* and *Firmicutes* might act as mycorrhiza helper bacteria [14,19,57]. Therefore, a holistic approach that comprehends arbuscular mycorrhiza as a tripartite symbiosis between plants, AMF, and bacteria should be considered in future investigations as a key to understanding changes in the functional and structural diversity of AMF in harsh habitats.

AMF communities were studied using the newly designed primers and a pipeline for high-throughput sequencing of LSU rDNA. We used SSU–ITS–LSU rDNA reference sequences to produce a reliable backbone phylogenetic tree for the phylogenetic placement of shorter AMF LSU rDNA sequences. We developed this approach in 2018, in parallel to Senés-Guerrero et al. [58] and Delavaux et al. [59,60], who showed that the phylogenetic placement of LSU rDNA AMF sequences on reference trees provides a more accurate taxonomic classification of the environmental sequences compared with blast-based methods. Unlike Senés-Guerrero et al. [58] and Delavaux et al. [59,60], we used LSU rDNA primers non-specific for Glomeromycota that provided an efficient amplification of “AMF size-specific” products on the template of low-quality DNA from the contaminated soil, followed by the selection of Glomeromycota amplicons based on electrophoretic resolution. The primers allowed a successful amplification using low-quality DNA extract from hydrocarbon-contaminated soil as a template, while other primers targeting longer regions failed. Using this approach, we were able to detect at higher levels of representation taxa that are generally not found dominant in the environmental studies of AMF communities. A pitfall of the approach was the high number of non-Glomeromycota sequences removed during the filtering of sequences, possibly due to inaccurate band excision from the gel. This problem could be easily solved by improving the parameters of the electrophoretic run. Nevertheless, the high number of sequences produced by the high-throughput sequencing guaranteed a high coverage of the AMF diversity.

## 4. Materials and Methods

### 4.1. Sampling and DNA Extraction

The sampling sites and sampling design for this study were the same as described by Malicka et al. [19]. In brief, samples of roots and soil contaminated with phenol and PAHs were collected from the shore of Kalina Pond located at Świętochłowice (50°16′49″ N, 18°55′38″ E). The control site was represented by the shore of Kokotek Pond II located at Lubliniec (50°37′14″ N, 18°43′46″ E). Soil cores with roots were collected from two dominant plant species, *Ph. australis* and *P. trivialis*. At each study site, twelve root and twelve soil samples were collected for each plant species. Overall, 48 root and 48 soil samples were available for analysis. Samples were stored at −20 °C before DNA extraction. Soil physicochemical analyses (phenol and PAH concentrations, soil granulometry, moisture content, SOM content, pH, and Ntot) and isolation of total DNA from roots and soil were performed as described previously by Malicka et al. [19]. 

### 4.2. Primer Design

In order to design a set of primers targeting the highly variable D2 region of the LSU rDNA subunit, a dataset of about 1850 fungal sequences was prepared (File S2). The sequences were retrieved from Genbank using a Primer Blast approach to select only those sequences covering the region between the ITS3 and the NDL22 primers [61,62]. The alignment of the dataset with MAFFT (https://mafft.cbrc.jp/alignment/server/, strategy = auto, accessed on 3 September 2018) [63] revealed an interesting characteristic: sequences belonging to Glomeromycota, Mucoromycota and Mortierellomycota possessed one to two insertions of variable length in the portion of the alignment close to the NDL22 primer site (Figure S10). This characteristic prompted the authors to test a PCR strategy with target specificity given mainly by the length of the amplicons instead of the annealing of the primers. FULF and FULR primers amplifying the D2 region of LSU rDNA sequences were designed on the conserved motifs of Glomeromycota sequences in the dataset alignment, with a maximum of three mismatches tolerated when not in the last five residues at the 3’ end (Table 3). The conserved motifs used for primer design were also shared by sequences belonging to taxa other than Glomeromycota. The primers were used to query GenBank using Primer-BLAST to target fungal and eukaryotic sequences (Files S3–S5). The Primer-BLAST output confirmed that FULF and FULR are universal fungal primers that enable the selection of Glomeromycota sequences from the other fungal taxa by the length of a PCR product. The length of the 33,602 Glomeromycota amplicons obtained in silico ranged from 336 bp (*Entrophospora infrequens*) to 446 bp (*Diversispora* sp.). Among almost 50,000 non-Glomeromycota fungal sequences screened, only 1100 exceeded 320 bp, 67% belonging to Mucoromycota/Mortierellomycota phyla, 15% to Zoopagomycota, and 11% to Chytridiomycota (File S6). When the primers were tested in Primer Blast vs. plants sequences, no amplicon >300 bp and <1000 bp was detected. Furthermore, several tested amplicons <300 bp that belonged to fungi were wrongly identified as plant species.

The second set of primers for nested PCR, including the adaptors for sequencing, was designed following the same criteria for specificity described above (Table 3).

Finally, both sets of primers were successfully tested vs. the curated database used for the phylogenetic analysis (database described in Section 4.4 Bioinformatic analysis).

### 4.3. PCR and Preparation of LSU rDNA Libraries for Sequencing

The first PCR reaction with the primers FULF and FULR was performed in a total volume of 25 µL with DreamTaq Polymerase (Thermo Fisher Scientific, Waltham, Massachusetts, USA), with the addition of 20 μg bovine serum albumin μL^−1^ and 3 mM MgCl_2_, in the T100 Thermal Cycler (Bio-Rad, Hercules, CA, USA), using the following conditions: 15 min at 95 °C, 34 cycles of 20 s denaturation at 95 °C, 30 s annealing at 56 °C, 45 s elongation at 72 °C, and final elongation for 5 min at 72 °C. The PCR products were resolved by electrophoresis in 3% agarose gel (Basica LE GQT Prona Agarose, Resolva GQT Prona Agarose; 1:1; *w*/*w*). The > 330 bp products, which were expected to contain Glomeromycota sequences, were selected by excision from the agarose gel, followed by purification with Wizard^®^ SV Gel and PCR Clean-Up System (Promega, Madison, Wisconsin, USA). The products were diluted (1:9) and used as a template for the second PCR reaction with the primers FULFN1ngs/FULFN2ngs–FULRNngs containing the Illumina adaptors. PCR reactions in a total volume of 50 µL were performed with DreamTaq Polymerase using the following thermal cycling conditions: 1 min at 95 °C, 34 cycles of 15 s at 95 °C, 30 s at 56 °C, 30 s at 72 °C, and 5 min final elongation at 72 °C. The attachment of dual indices was performed following the 16S Metagenomic Sequencing Library Preparation guide of Illumina [64] with some modifications, as described in File S7. The libraries were analyzed with Agilent 2200 TapeStation System with D1000 ScreenTape kit (Agilent Technologies, Santa Clara, CA, USA) and quantified using the Invitrogen QubitTM fluorometer with the QubitTM dsDNA HS Assay kit (Thermo Fisher Scientific, Waltham, MA, USA). Ninety-six libraries were normalized, pooled, and pair-end sequenced (2 × 250 bp) using MiSeq with MiSeq Reagent Kit v2 (Illumina, San Diego, CA, USA) with the output of ~100,000 reads per library. The qualitative and quantitative analyzes and sequencing were performed by Bionanopark Ltd. (Łódź, Poland). 

### 4.4. Bioinformatic Analysis

A reference sequence dataset for phylogenetic affiliation of the NGS sequences was prepared. It comprised all the Glomeromycota species described that contained the LSU rDNA sequences delimited by the primers used in the present study. Overall, the 629 sequences in the dataset represented 45 genera and 196 species from all the orders and families recognized in Glomeromycota. Additional undescribed taxa, particularly in the recently described family of *Polonosporaceae* [48], were also represented. About 75% of the sequences covered the SSU–ITS–LSU rDNA portion. The dataset was aligned with MAFFT (strategy = auto), trimmed in MEGA X 10.1.7 [65] at the annealing sites of the SSUmCf and LSUmBr primers [27], and re-aligned with MAFFT (strategy = E-INS-i) (File S8). The alignment was divided into five partitions (18S, ITS1, 5.8S, ITS2, 28S) and used to infer a maximum likelihood (ML) phylogenetic tree with RAxML-HPC2 on XSEDE 8.2.12 [66] at CIPRES Science Gateway [67] with 1000 bootstrap iterations and a GTRGAMMAI nucleotide substitution model (Figure S11, File S9). The tree was visually inspected with the Archaeopteryx tree viewer [68].

The sequences were processed with Mothur 1.39 [69] software using the command batch provided in the File S10. The Glomeromycota sequences filtered for the expected size and chimeras were eventually clustered with a 0.02 cutoff using a Vsearch DGC method [70]. The representative sequences were aligned to the LSU rDNA reference sequence dataset in MAFFT using the FFT-NS-2 method and 200PAM/k = 2 scoring matrix. The alignment was used as an input for RAxML-EPA [71] to place the sequences on the reference ML tree (Figure S11, File S9) using a GTRGAMMAI nucleotide substitution model. The jplace output was used in GAPPA v0.8.0 [72] for the placement mass (likelihood weight ratio–LWR) accumulation of each sequence upwards the reference tree with threshold = 0.9 and their taxonomic annotation (reference taxonomy file provided in the File S11). Sequences assigned to the same species clade were merged and served as taxonomic units for further analysis. The shared file from Mothur (File S12) was modified accordingly (File S13).

### 4.5. Analysis of AMF Communities Diversity

The analysis of the α-diversity of AMF communities was performed in Mothur 1.39 based on the modified shared file (File S13). The comparison between communities was supported by statistical analysis using Past 4.03 [73]. Rarefaction curves and Good’s coverage index were calculated to evaluate the sequencing quality. To overcome the differences in the number of sequences between samples, the α-diversity estimator (the number of observed species, Chao1, ACE, Boneh’s, the Shannon index, and the Shannon index-based measure of evenness) were calculated from the subsample of 100 sequences with 1000 iterations. To determine the effect of site contamination, plant species and source of DNA on the α-diversity of AMF communities, PERMANOVA (*p* < 0.05) was performed with α-diversity estimators as variables, calculating Euclidean distances. Significant differences in the values of each diversity estimator between the communities were indicated with the Kruskal–Wallis test (*p* < 0.05), followed by Mann–Whitney post-hoc pairwise comparisons with Bonferroni correction. 

The analysis of β-diversity of the AMF communities was based on two approaches: considering the dissimilarities in species composition and phylogenetic placements. The species-based analysis was performed using tools implemented in Mothur 1.39. The Yue and Clayton measure of dissimilarity in species proportion [74] was used to calculate the distance matrix to perform NMDS, AMOVA and HOMOVA. The Spearman’s correlation between the relative abundance of each species, soil physicochemical parameters, and the axes was calculated to determine which AMF species and soil physicochemical parameters defined the distribution of the AMF communities along the NMDS axes. The Metastats method [75] was used to determine the significant differences in the AMF species abundances between the AMF communities associated with different sites, plants, and roots and soil. The DMM model [76] was implemented to partition the analyzed AMF communities into metacommunities and to recognize which AMF species defined the partitioning.

The phylogeny-based analysis of the β-diversity of the AMF communities was performed using GAPPA v0.8.0. The ‘heat tree’ command was used to present the placement masses on tree edges for each of the analyzed AMF communities [72]. The Edge PCA was performed based on the imbalance of placements across the edges of the phylogenetic tree [72,77]. The imbalance k-means clustering was run with k = 8 (number of the analyzed AMF communities: two sites × two plant species × two matrices—roots and soil) in order to group the communities according to the Euclidean distance of the edge imbalances of the samples [72,78]. Spearman’s correlation coefficients were calculated between the placement masses on tree edges and physicochemical features of soil [72,78]. The placement-factorization method was used to implement the GLM to analyze the relationship between the abundance of placements on the edges of the tree and the value of soil contamination and physicochemical features [72,78,79].

### 4.6. Accession Numbers

Raw sequence data have been deposited in the SRA NCBI database under the BioProject accession number PRJNA864584.

## 5. Conclusions

A comparison of the diversity of the root and soil AMF communities associated with different plant species (*P. trivialis* and *Ph. australis*) from a site contaminated with phenol and PAHs and an uncontaminated control site has revealed that soil contamination had a very strong influence on the richness, abundance, and composition of the AMF communities. Biotic factors, such as plant host identity and biotope (roots/soil), were of lower importance in the shaping of the AMF diversity under hydrocarbon contamination; however, they were significant drivers of AMF diversity at the uncontaminated site in the absence of anthropogenic pressure. The presence of phenol and PAHs decreased the species richness of AMF communities and favored the dominance of AMF from the families *Glomeraceae* and *Paraglomeraceae*. *Glomeraceae* were represented mainly by two genera, *Rhizoglomus* and *Funneliformis*, whose functional traits and life strategies make them stress-tolerant. These AMF might be promising candidates for designing mycorrhizal inocula for supporting the phytoremediation of hydrocarbon-contaminated soils. They could be considered as a tool to support plant growth and stimulate the activity of hydrocarbon-degrading bacteria. Plants growing at the uncontaminated site were inhabited by diverse species of *Glomeraceae* and early divergent AMF lineages (*Paraglomeraceae* and *Polonosporaceae*) that might provide fewer nutrients to a host plant than phylogenetically younger AMF lineages. The high abundance of early diverging AMF might be explained by the use of high-resolution primers detecting lineages such as *Paraglomeracae* that have not been recognized by previously used 18S rDNA primers.

The AMF communities were characterized by the NGS of the LSU rDNA with new primers which prioritize the coverage of all families within Glomeromycota over specificity. This approach allowed easy band size-based separation of Glomeromycota sequences from other non-target fungal sequences. Compared with other short-read NGS strategies based mostly on the SSU rDNA and ITS molecular markers, the use of the LSU rDNA provided complete coverage of Glomeromycota and better taxonomic resolution of closely-related species, avoiding the discrimination or exclusion of some AMF families such as *Paraglomeraceae* from the communities.

## Figures and Tables

**Table 1:** The values of α-diversity estimators (mean ± SD) of the roots and soil AMF communities associated with *P. trivialis* (Pt) and *Ph. australis* (Pa) collected from the site contaminated with phenol and PAHs and the uncontaminated site. *p*-values from the permutational analysis of variance (PERMANOVA) indicate significant differences between the AMF communities associated with different sites, plants, or matrices (roots/soil). *p*-values from the Kruskal–Wallis test indicate significant differences between the AMF communities for each estimator (species observed, Chao1, ACE, Shannon Index, Evenness Index). Significant post-hoc pairwise Mann–Whitney U comparisons (*p* < 0.05) between the communities for each estimator are indicated by different superscript letters.

Site (*p* < 0.001)	Plant (*p* = 0.232)	Sample (*p* = 0.013)	Species (*p* < 0.001)	Chao1 (*p* < 0.001)	ACE (*p* < 0.001)	Boneh	Shannon Index (*p* < 0.001)	Evenness Index (*p* = 0.052)
Cont.	Pt	Root	5 ± 2 ^b^	5 ± 2 ^b^	6 ± 5 ^b^	0 ± 0	0.71 ± 0.38 ^c^	0.48 ± 0.19
		Soil	7 ± 2 ^b^	8 ± 3 ^b^	10 ± 5 ^ab^	1 ± 0	1.15 ± 0.36 ^bc^	0.63 ± 0.12
	Pa	Root	4 ± 2 ^b^	4 ± 2 ^b^	4 ± 3 ^b^	0 ± 0	0.77 ± 0.63 ^bc^	0.60 ± 0.26
		Soil	5 ± 2 ^b^	5 ± 3 ^b^	6 ± 4 ^b^	0 ± 0	0.84 ± 0.54 ^bc^	0.65 ± 0.24
Uncont.	Pt	Root	11 ± 2 ^a^	13 ± 3 ^a^	14 ± 3 ^a^	1 ± 1	1.77 ± 0.24 ^a^	0.74 ± 0.07
		Soil	12 ± 4 ^a^	16 ± 6 ^a^	20 ± 6 ^a^	2 ± 1	1.57 ± 0.51 ^ab^	0.64 ± 0.16
	Pa	Root	8 ± 1 ^ab^	9 ± 2 ^b^	12 ± 4 ^ab^	1 ± 0	1.36 ± 0.21 ^b^	0.68 ± 0.08
		Soil	9 ± 3 ^ab^	11 ± 5 ^ab^	18 ± 9 ^a^	1 ± 0	1.42 ± 0.44 ^ab^	0.66 ± 0.14

**Table 2:** The results of the non-parametric analysis of the homogeneity of molecular variance (HOMOVA) and AMOVA indicating the significant differences in the structure of soil (S) and root (R) AMF communities associated with *P. trivialis* (Pt) and *Ph. australis* (Pa) collected from the contaminated (K) and uncontaminated (C) sites.

	Variance		AMOVA	HOMOVA
	Group 1	Group 2		
Site (K/C)	0.311	0.336	*p* < 0.001	*p* = 0.240
Plant (Pt/Pa)	0.357	0.341	*p* < 0.001	*p* = 0.494
Sample (R/S)	0.381	0.346	*p* = 0.012	*p* = 0.122
K_Pa/K_Pt	0.302	0.383	*p* = 0.119	*p* = 0.169
C_Pa/C_Pt	0.277	0.229	*p* < 0.001	*p* = 0.059
K_Pt/C_Pt	0.277	0.302	*p* < 0.001	*p* = 0.264
K_Pa/C_Pa	0.229	0.383	*p* < 0.001	*p* = 0.007

**Table 3:** List of primers designed to amplify the D2 fragment of fungal LSU rDNA.

Primer	Sequences of Primers (5′–3′)	Position in *Rhizophagus irregularis* DAOM197198 Genome (BDIQ01000050)
FULF (forward)	GTGAAATTGTTGAAAGGGAAACG	2,474,826
FULR (reverse)	CCTTGGTCCGTGTTTCAAGAC	2,475,206
FULFN1ngs (forward)	* AGGGAAACGATTGAAGCCAGTC	2,478,820
FULFN2ngs (forward)	* GAAAGGGAAACGATTGAAGTCAGT	2,478,817
FULRNngs (reverse)	** CGTGTTTCAAGACGGGTCGT	2,475,199

The sequences of adapters: * TCGTCGGCAGCGTCAGATGTGTATAAGAGACAG, ** GTCTCGTGGGCTCGGAGATGTGTATAAGAGACAG.

## Data Availability

The data presented in this study are available in Table S3. Raw sequences data are publicly available in the SRA NCBI database under the accession number PRJNA864584.

## Supplementary Materials

The following supporting information can be downloaded at: https://www.mdpi.com/article/10.3390/ijms232012585/s1.
