# Supplementary material for: Phenol and Polyaromatic Hydrocarbons Are Stronger Drivers Than Host Plant Species in Shaping the Arbuscular Mycorrhizal Fungal Component of the Mycorrhizosphere
Source: Int J Mol Sci. 2022 Oct 20;23(20):12585. doi: 10.3390/ijms232012585 (PMC9604154; doi:10.3390/ijms232012585)
Supplement: Supplementary file 1 [file ijms-23-12585-s001.zip › Figure S1.pdf]

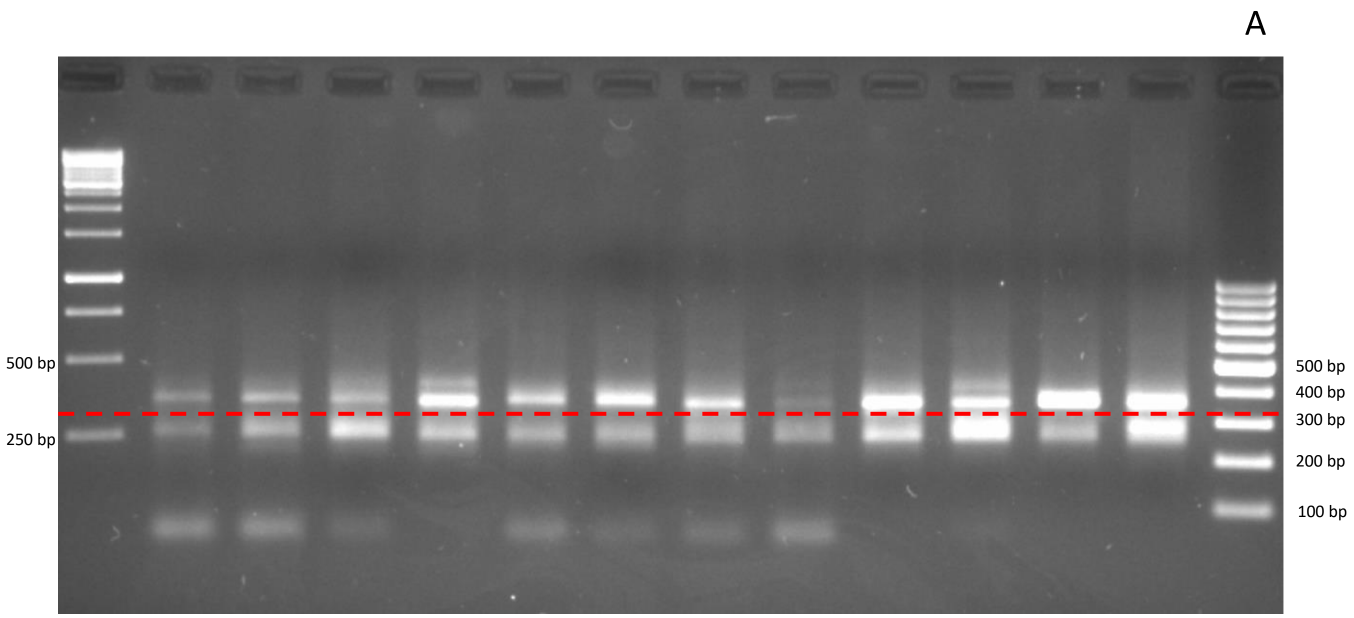

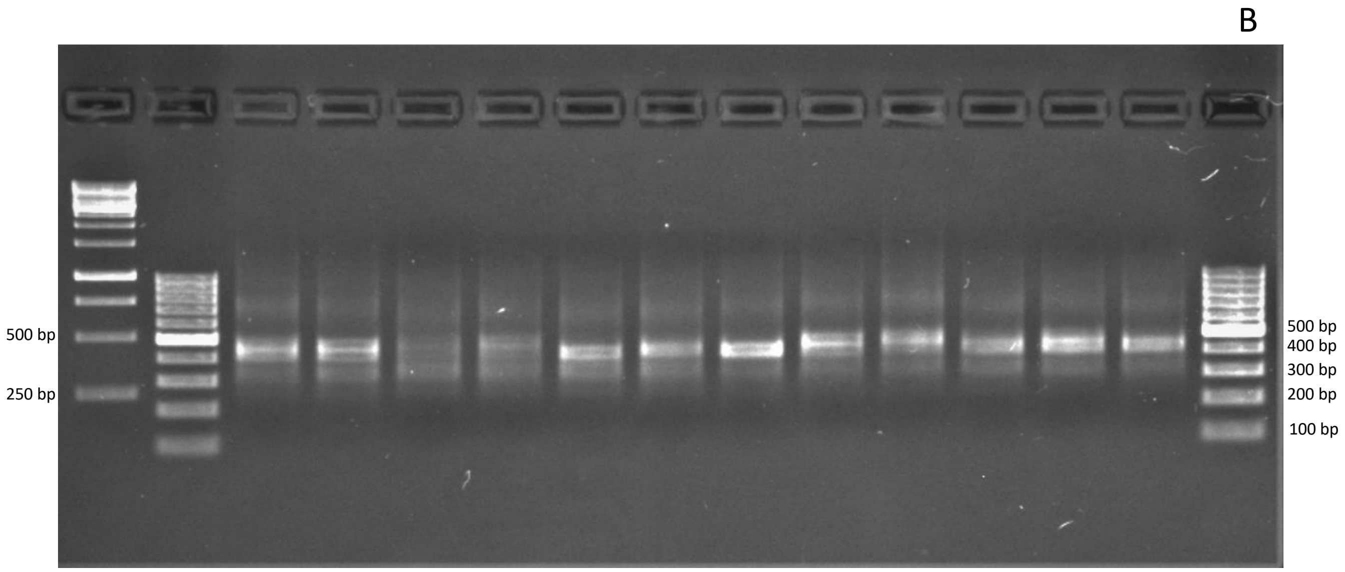

Figure S1. The products of PCR reaction with FULF and FULR primers resolved by agarose electrophoresis. The red dashed line separate the LSU rDNA amplicons of Glomeromycota (>330 bp, above the line) and the amplicons of Basidiomycota and Ascomycota (below the line) (A). The products of PCR reaction with FULFN1ngs/FULFN2ngs – FULRNngs primers with Illumina adaptors (B).
