# Supplementary material for: Phenol and Polyaromatic Hydrocarbons Are Stronger Drivers Than Host Plant Species in Shaping the Arbuscular Mycorrhizal Fungal Component of the Mycorrhizosphere
Source: Int J Mol Sci. 2022 Oct 20;23(20):12585. doi: 10.3390/ijms232012585 (PMC9604154; doi:10.3390/ijms232012585)
Supplement: Supplementary file 1 [file ijms-23-12585-s001.zip › Figure S11.pdf]

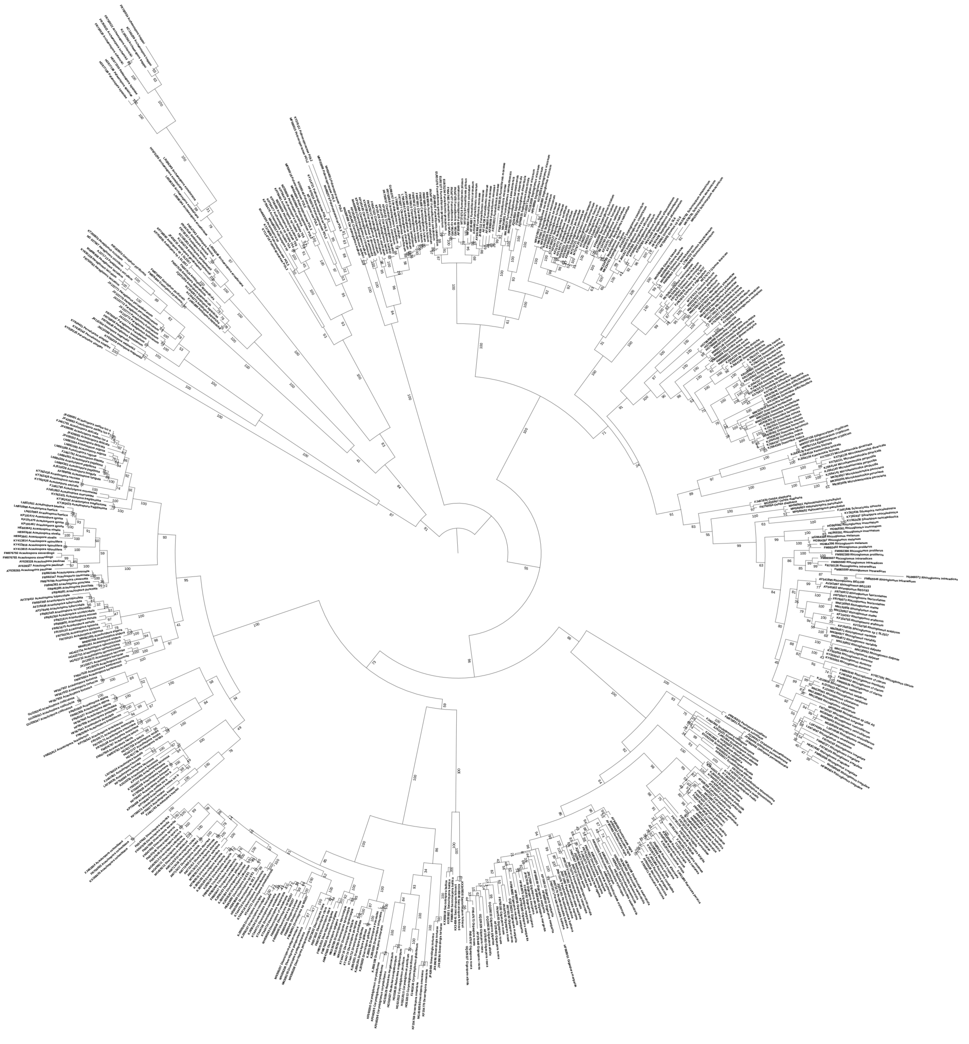

Figure S11. The reference phylogenetic tree constructed with RAxML-HPC2 (Stamatakis, 2014) with 1000 bootstrap iterations and GTRGAMMAI nucleotide substitution model using the alignment of 629 LSU rDNA sequences of Glomeromycota. The tree was visualized using iTOL.
