# Supplementary material for: Phenol and Polyaromatic Hydrocarbons Are Stronger Drivers Than Host Plant Species in Shaping the Arbuscular Mycorrhizal Fungal Component of the Mycorrhizosphere
Source: Int J Mol Sci. 2022 Oct 20;23(20):12585. doi: 10.3390/ijms232012585 (PMC9604154; doi:10.3390/ijms232012585)
Supplement: Supplementary file 1 [file ijms-23-12585-s001.zip › Figure S2.pdf]

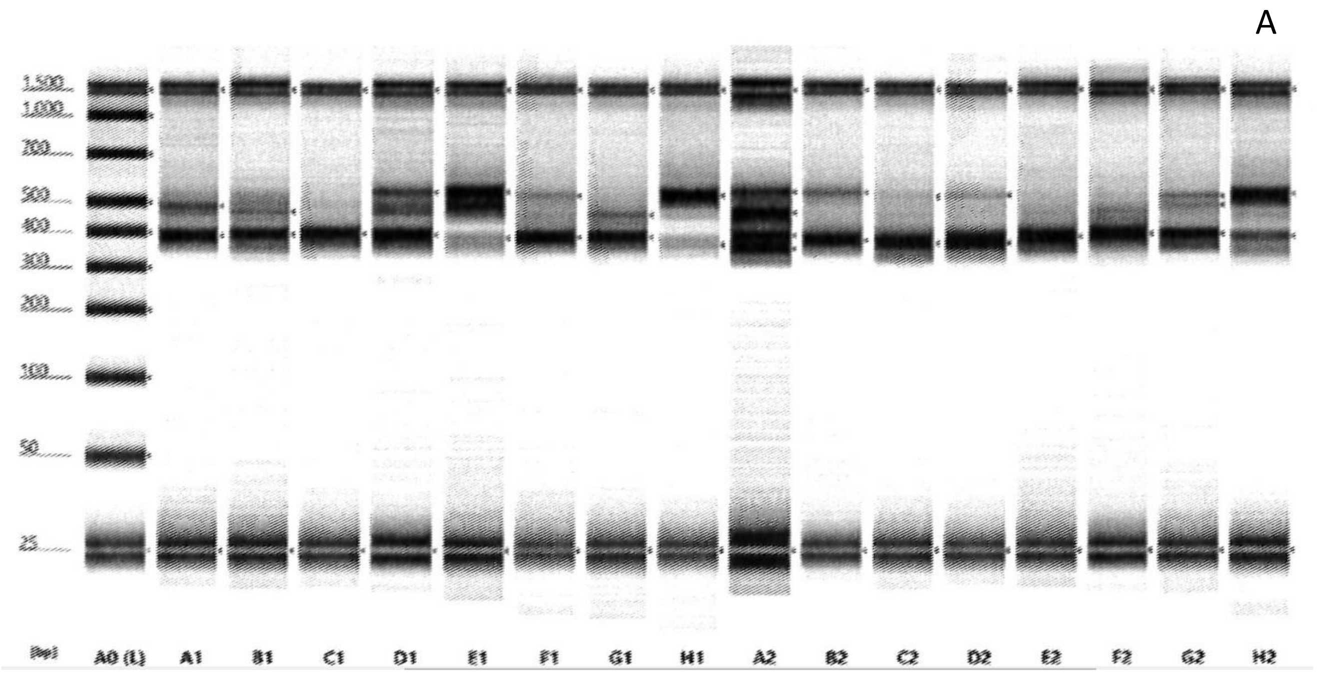

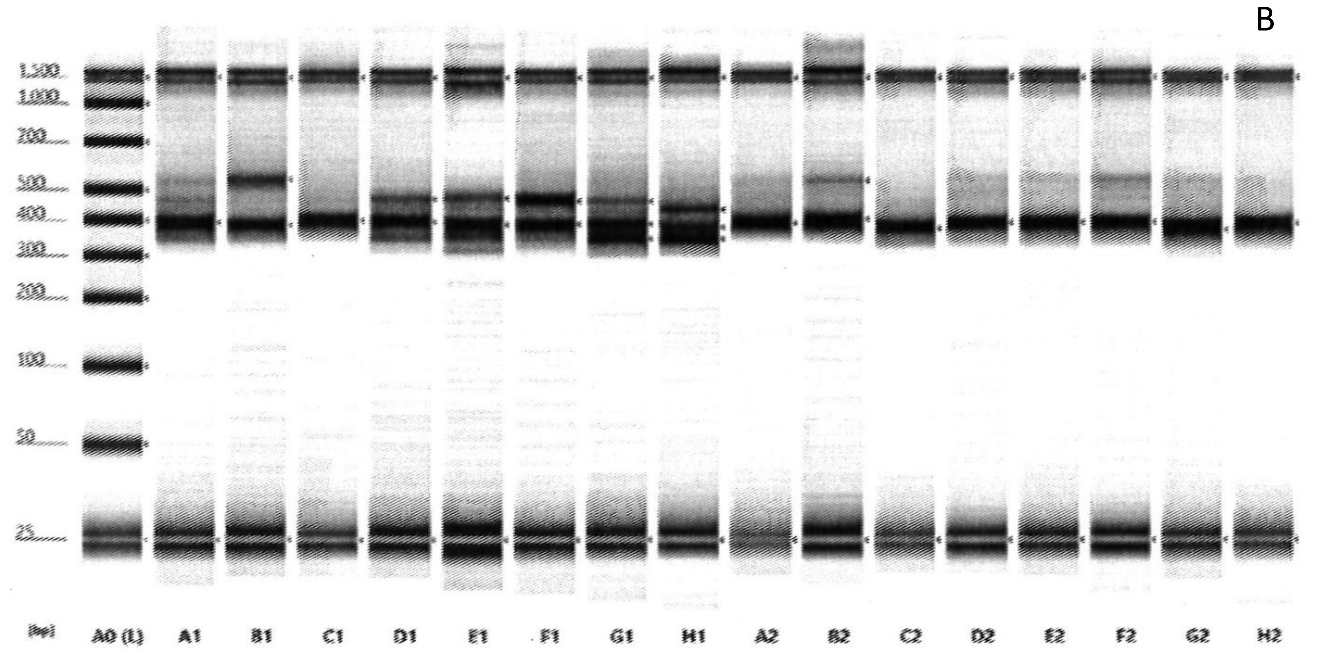

Figure S2. The results of electrophoretic analysis of the LSU rDNA libraries of *Glomeromycota* on Tape Station 2200. Samples corresponding to the gel pictures in Supplementary file 15 are marked as E2 – H2 in the gel A and A1 – H1 in the gel B. 25 bp and 1500 bp bands are molecular weight markers.
