# Supplementary material for: Phenol and Polyaromatic Hydrocarbons Are Stronger Drivers Than Host Plant Species in Shaping the Arbuscular Mycorrhizal Fungal Component of the Mycorrhizosphere
Source: Int J Mol Sci. 2022 Oct 20;23(20):12585. doi: 10.3390/ijms232012585 (PMC9604154; doi:10.3390/ijms232012585)
Supplement: Supplementary file 1 [file ijms-23-12585-s001.zip › Figure S3.pdf]

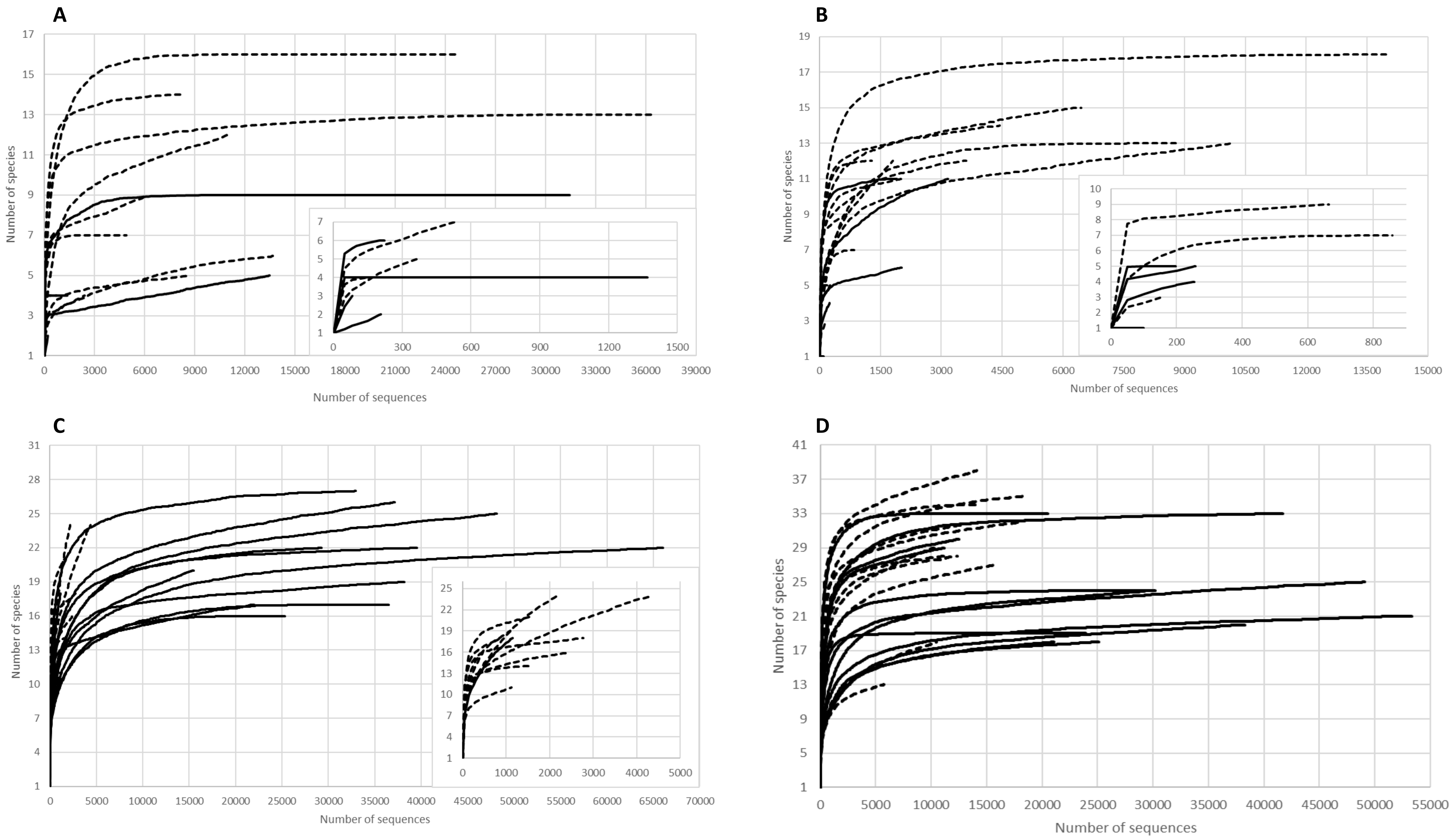

Figure S3. Rarefaction curves obtained for the AMF communities associated with *P. trivialis* (dashed lines) and *Ph. australis* (solid lines). Rarefaction curves for root and soil AMF communities at the contaminated site are presented on graphs A and B, respectively. Rarefaction curves for root and soil AMF communities at the uncontaminated site are presented on graphs C and D, respectively.
