# Supplementary material for: Phenol and Polyaromatic Hydrocarbons Are Stronger Drivers Than Host Plant Species in Shaping the Arbuscular Mycorrhizal Fungal Component of the Mycorrhizosphere
Source: Int J Mol Sci. 2022 Oct 20;23(20):12585. doi: 10.3390/ijms232012585 (PMC9604154; doi:10.3390/ijms232012585)
Supplement: Supplementary file 1 [file ijms-23-12585-s001.zip › Figure S4.pdf]

S_K_Pa

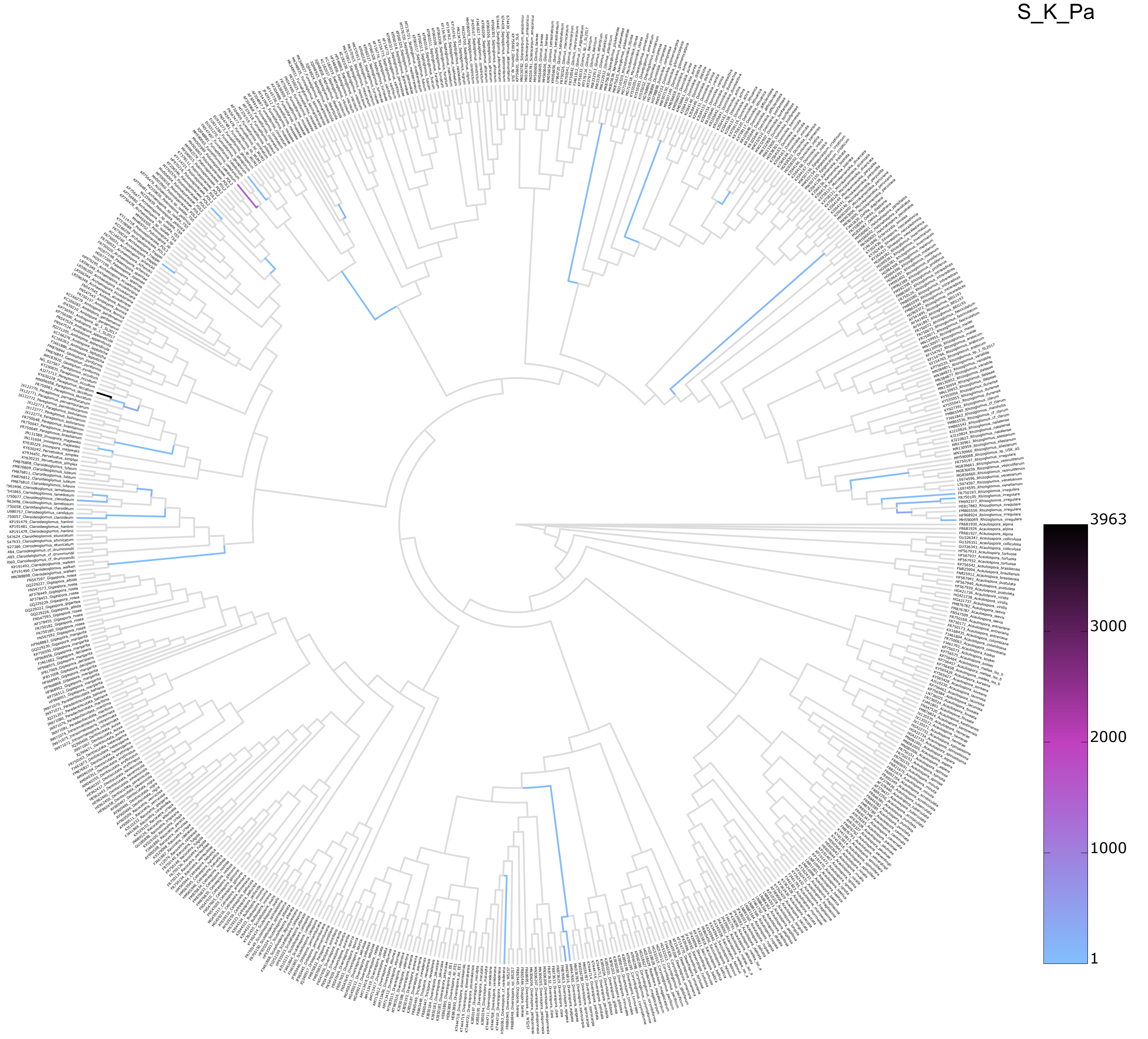

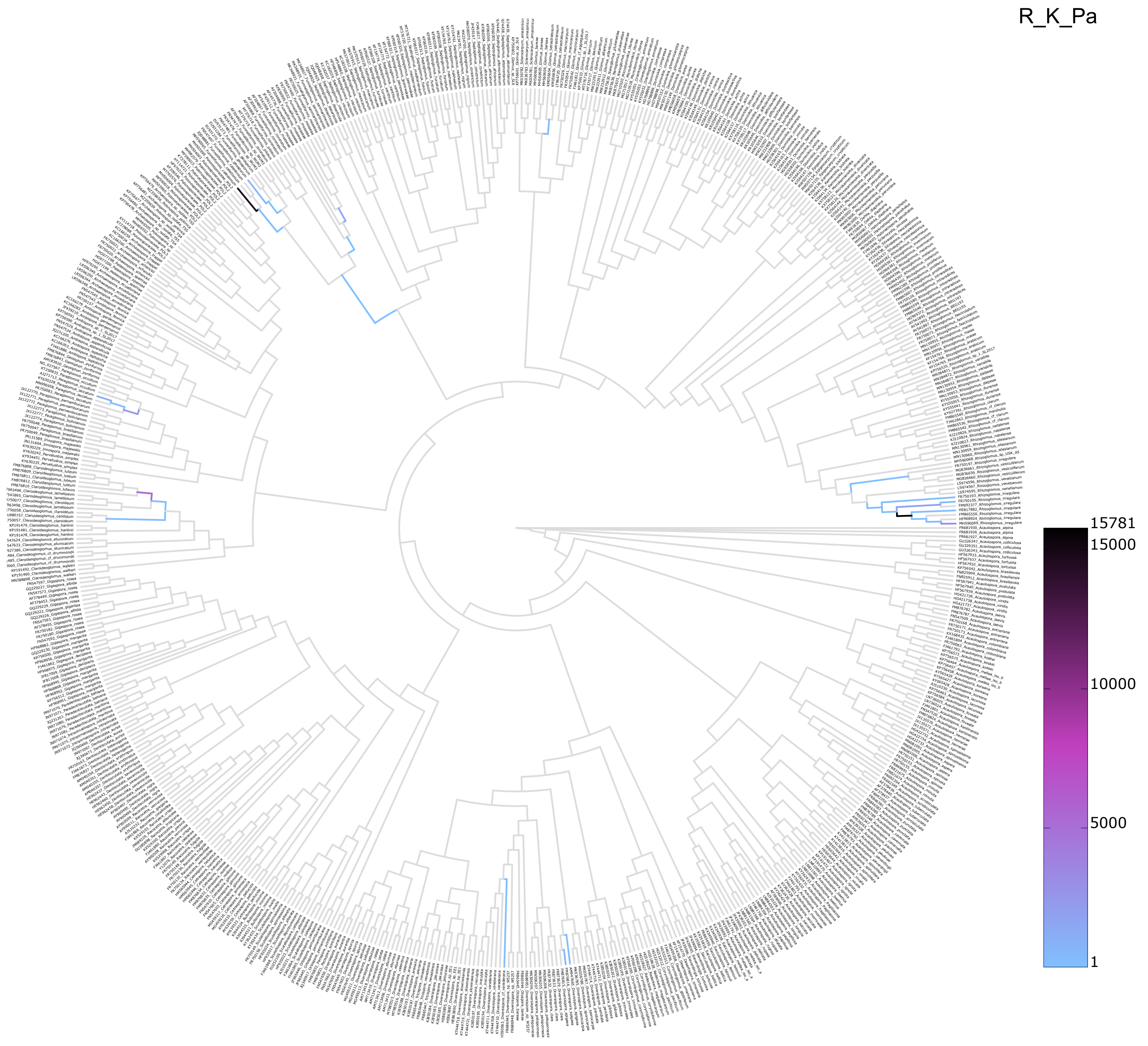
R_K_Pa
15781
15000
10000
5000
1

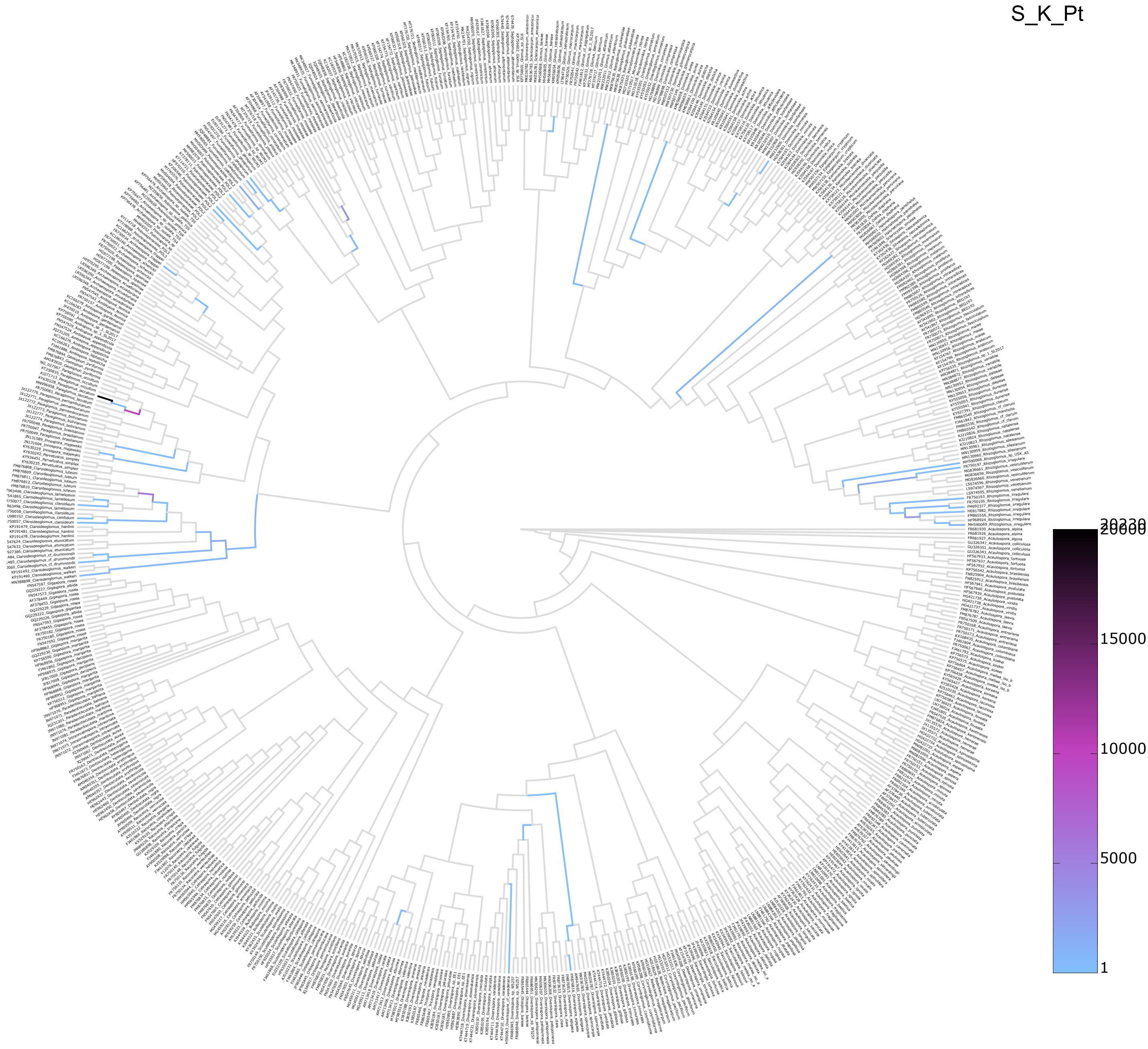
S_K_Pt
20000
15000
10000
5000
1

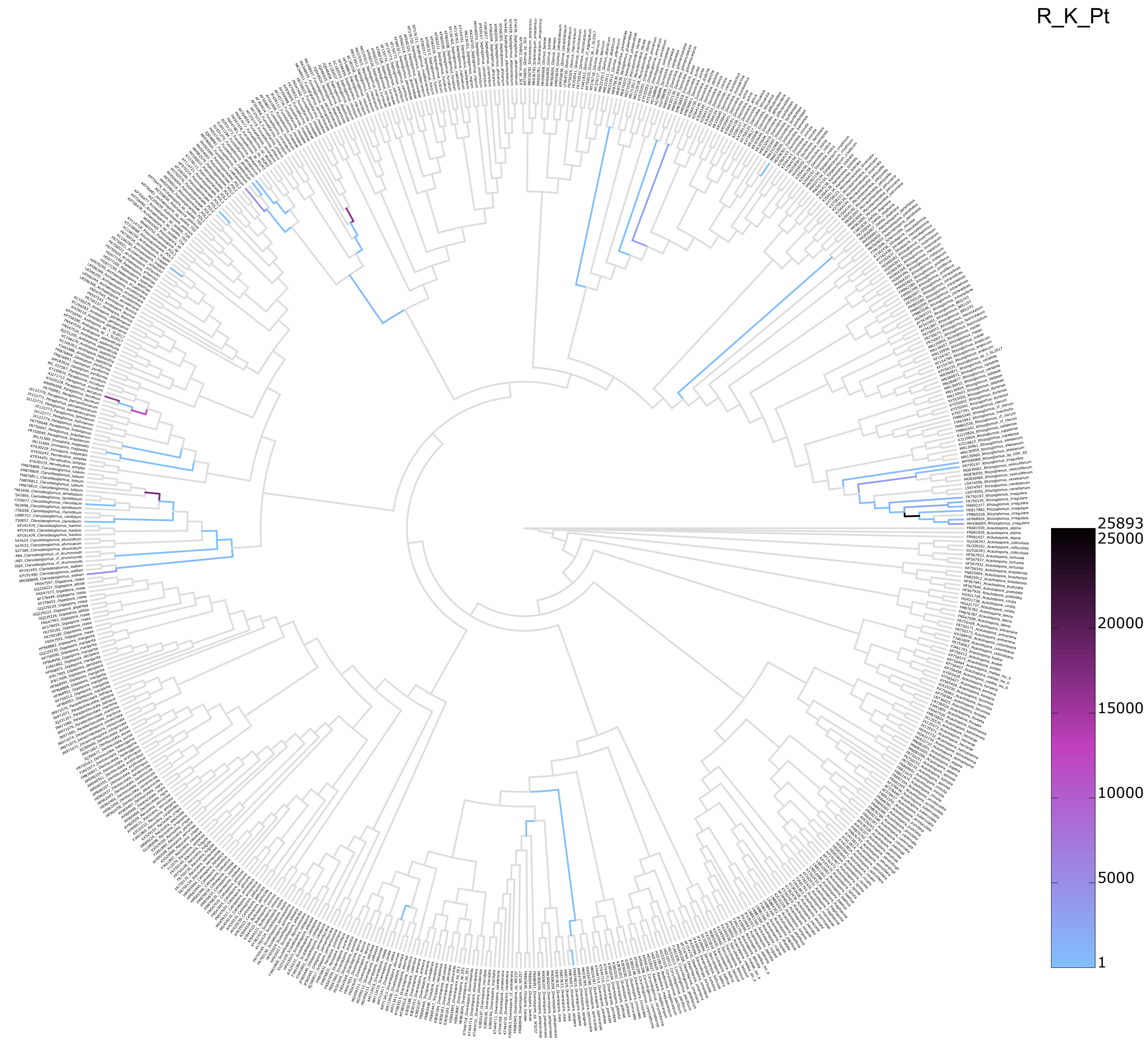
R_K_Pt
25893
25000
20000
15000
10000
5000
1

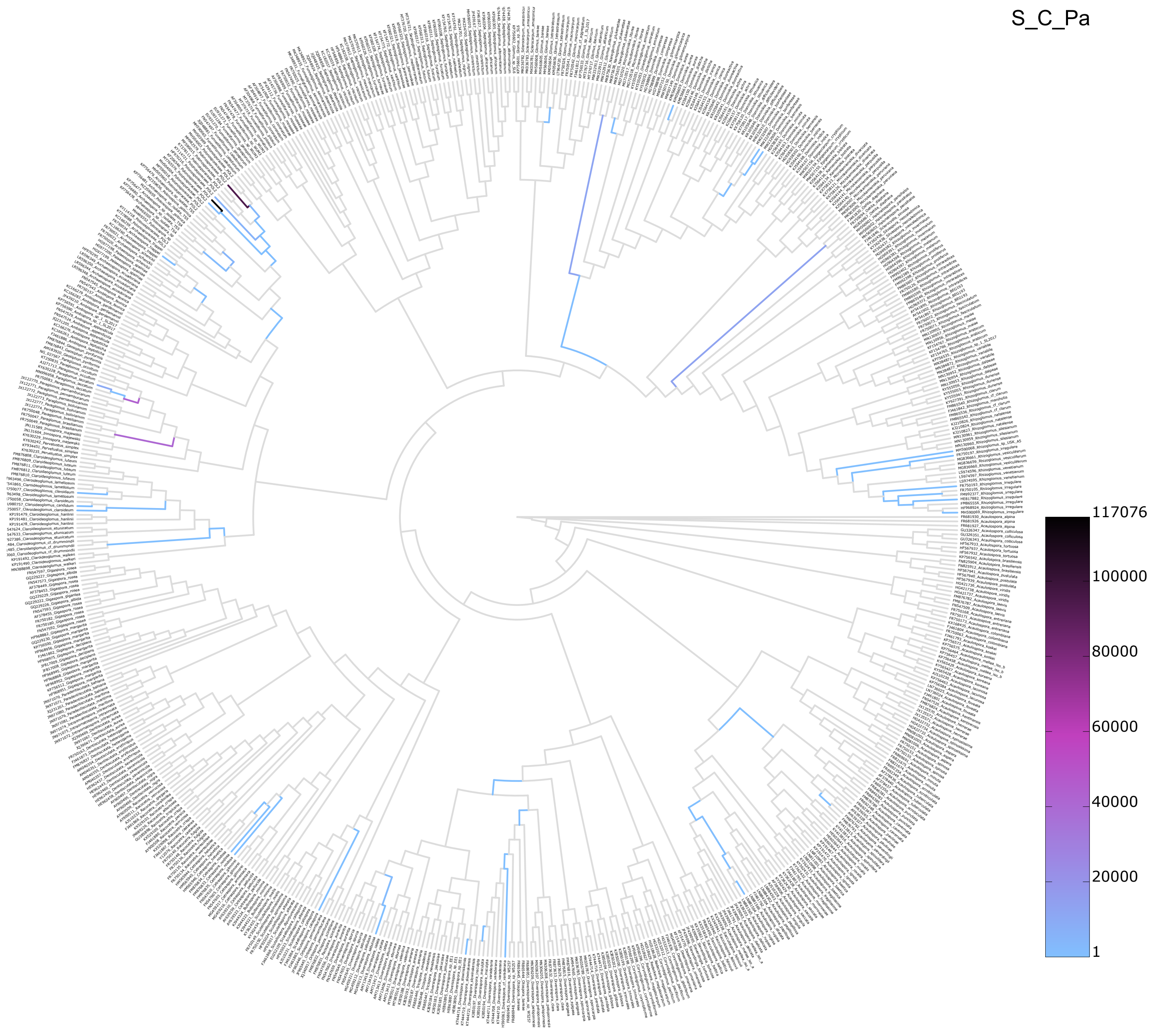

S_C_Pa
117076
100000
80000
60000
40000
20000
1

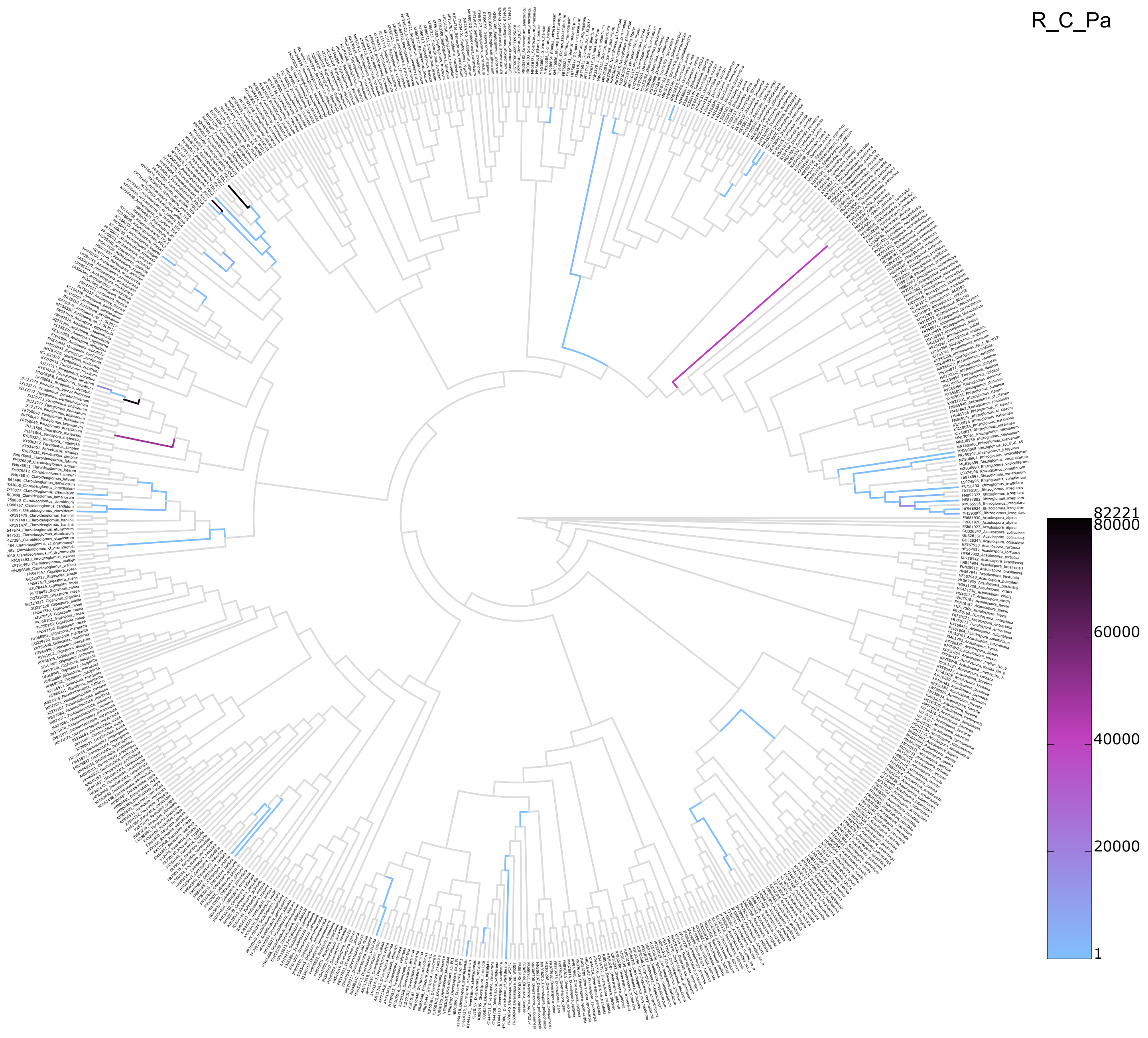
R_C_Pa
82221
80000
60000
40000
20000
1

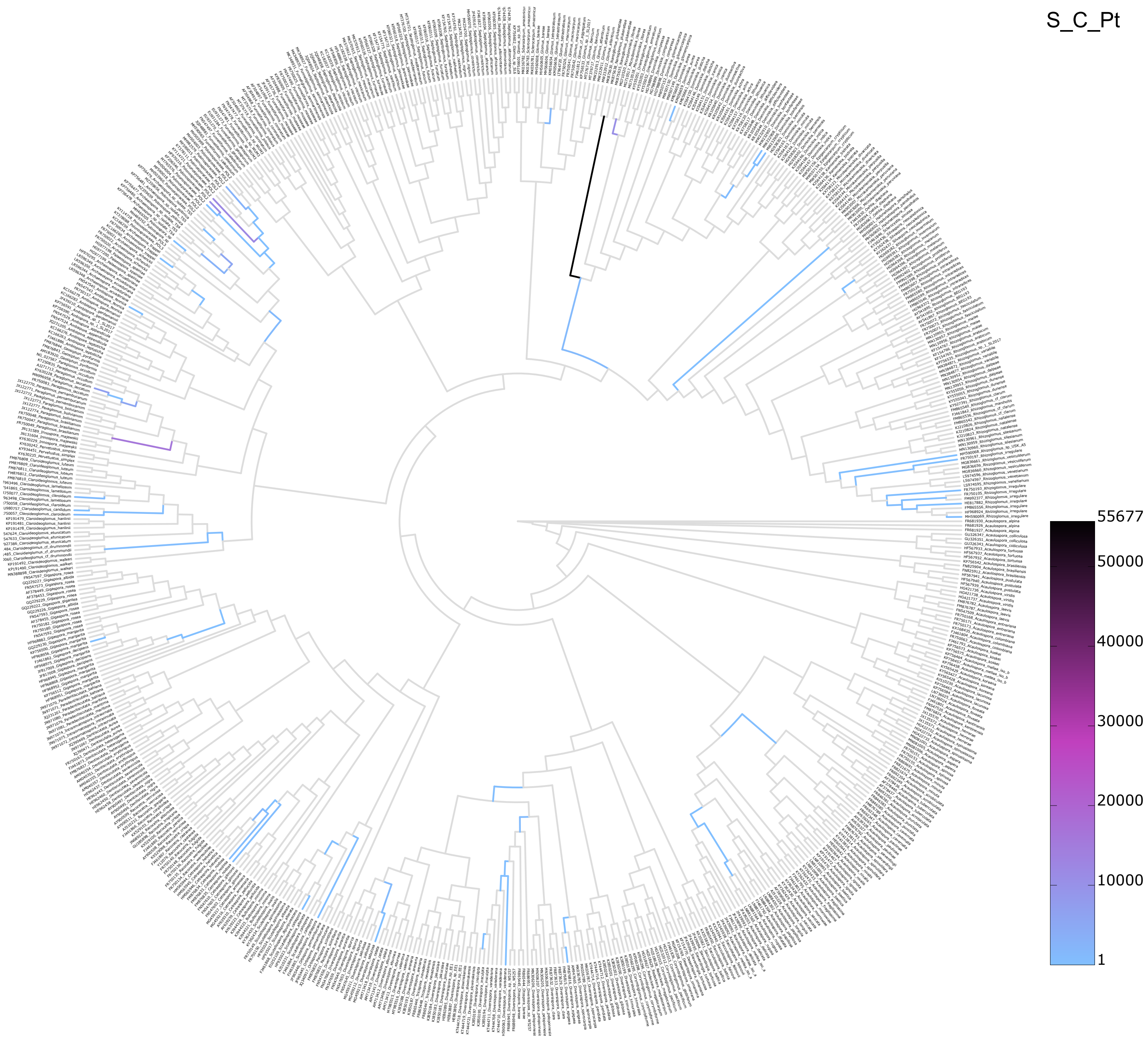

S_C_Pt
55677
50000
40000
30000
20000
10000
1

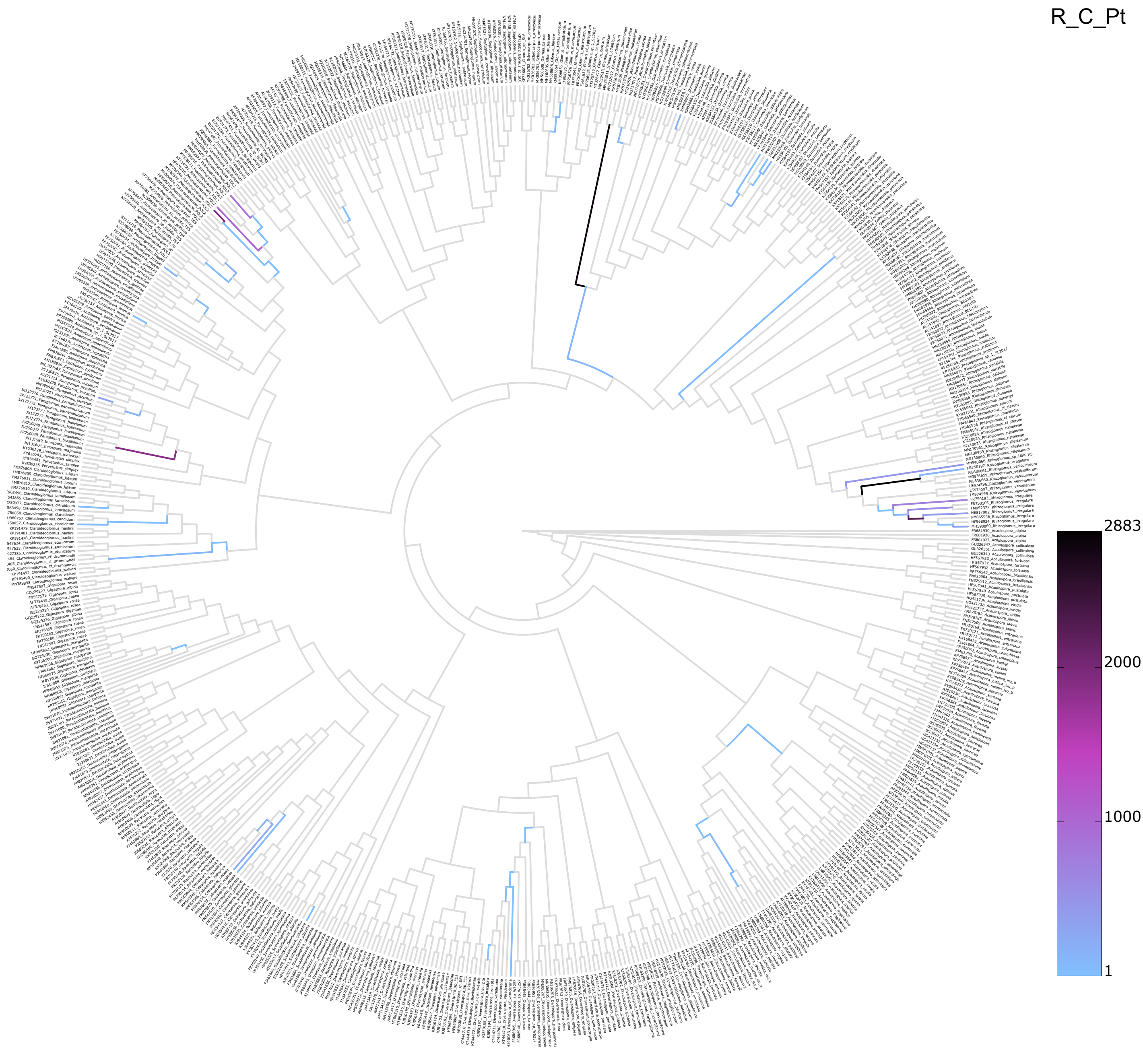

Figure S4. Heat-trees with edges coloured according to the placement mass of the root (R) and soil (S) AMF communities associated with P. trivialis (Pt) and Ph. australis (Pa) at the site contaminated with phenol and PAHs (K) and the uncontaminated site (C).
