# Supplementary material for: Phenol and Polyaromatic Hydrocarbons Are Stronger Drivers Than Host Plant Species in Shaping the Arbuscular Mycorrhizal Fungal Component of the Mycorrhizosphere
Source: Int J Mol Sci. 2022 Oct 20;23(20):12585. doi: 10.3390/ijms232012585 (PMC9604154; doi:10.3390/ijms232012585)
Supplement: Supplementary file 1 [file ijms-23-12585-s001.zip › Figure S5.pdf]

A

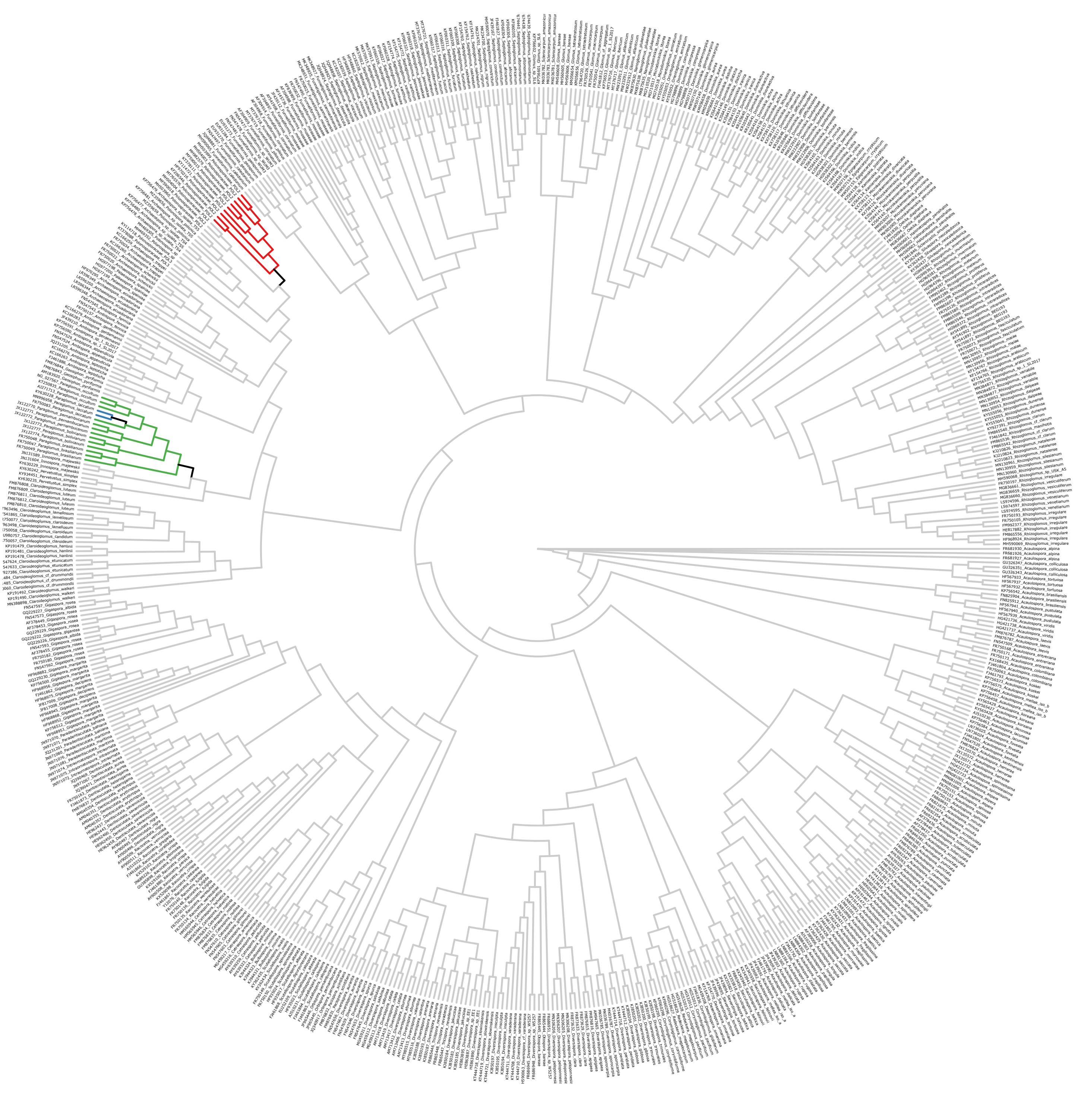

B

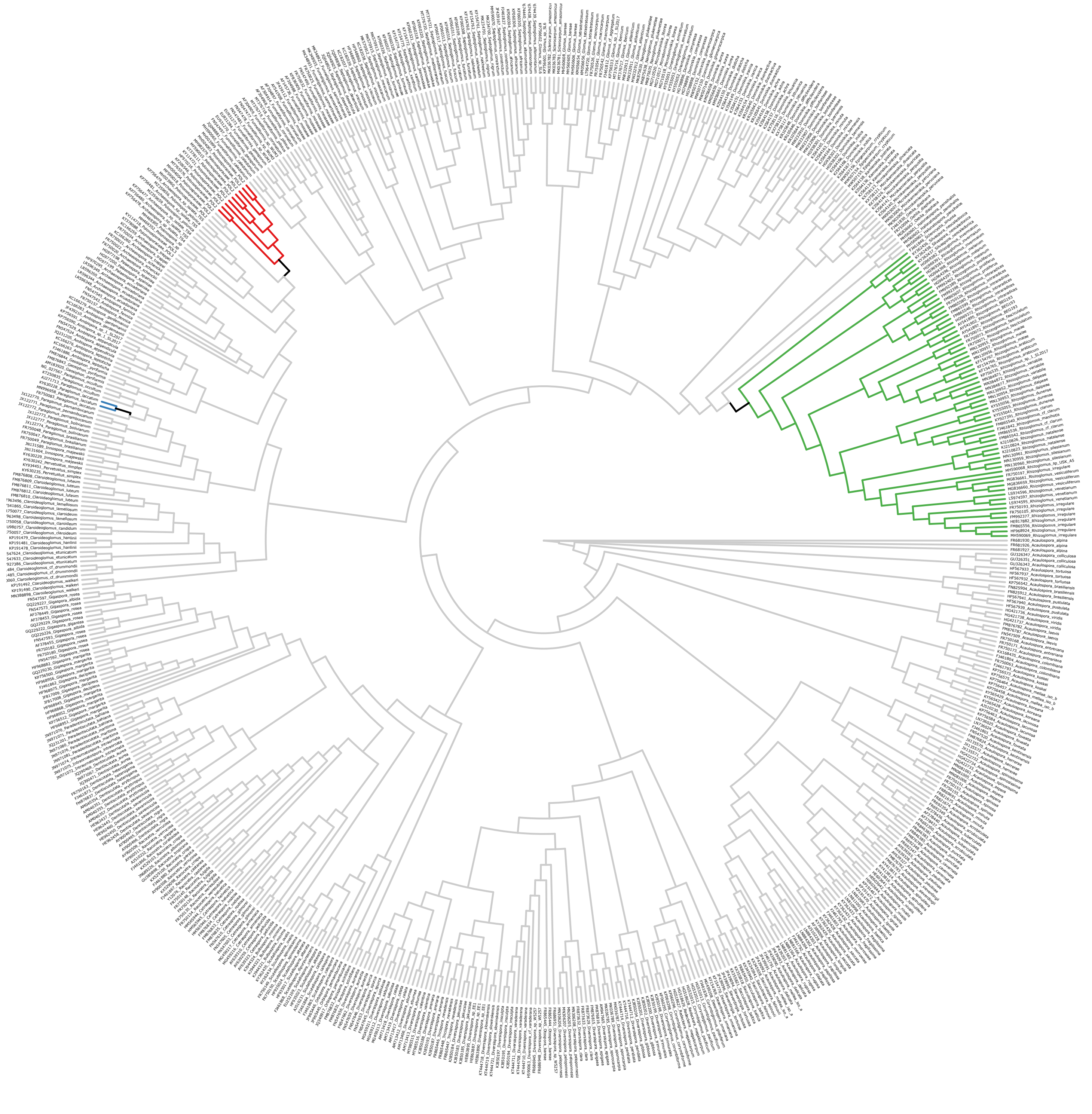

Figure S5. Trees showing the clades that are split by each phylofactor: F1 (red), F2 (blue), F3 (green) in relation to phenol and PAH contamination (A) and all of the examined soil physicochemical features : phenol, PAHs, SOM, Ntot, pH water, pH KCl (B). The winning edges for each factor are bold.
