# Supplementary material for: Phenol and Polyaromatic Hydrocarbons Are Stronger Drivers Than Host Plant Species in Shaping the Arbuscular Mycorrhizal Fungal Component of the Mycorrhizosphere
Source: Int J Mol Sci. 2022 Oct 20;23(20):12585. doi: 10.3390/ijms232012585 (PMC9604154; doi:10.3390/ijms232012585)
Supplement: Supplementary file 1 [file ijms-23-12585-s001.zip › Figure S6.pdf]

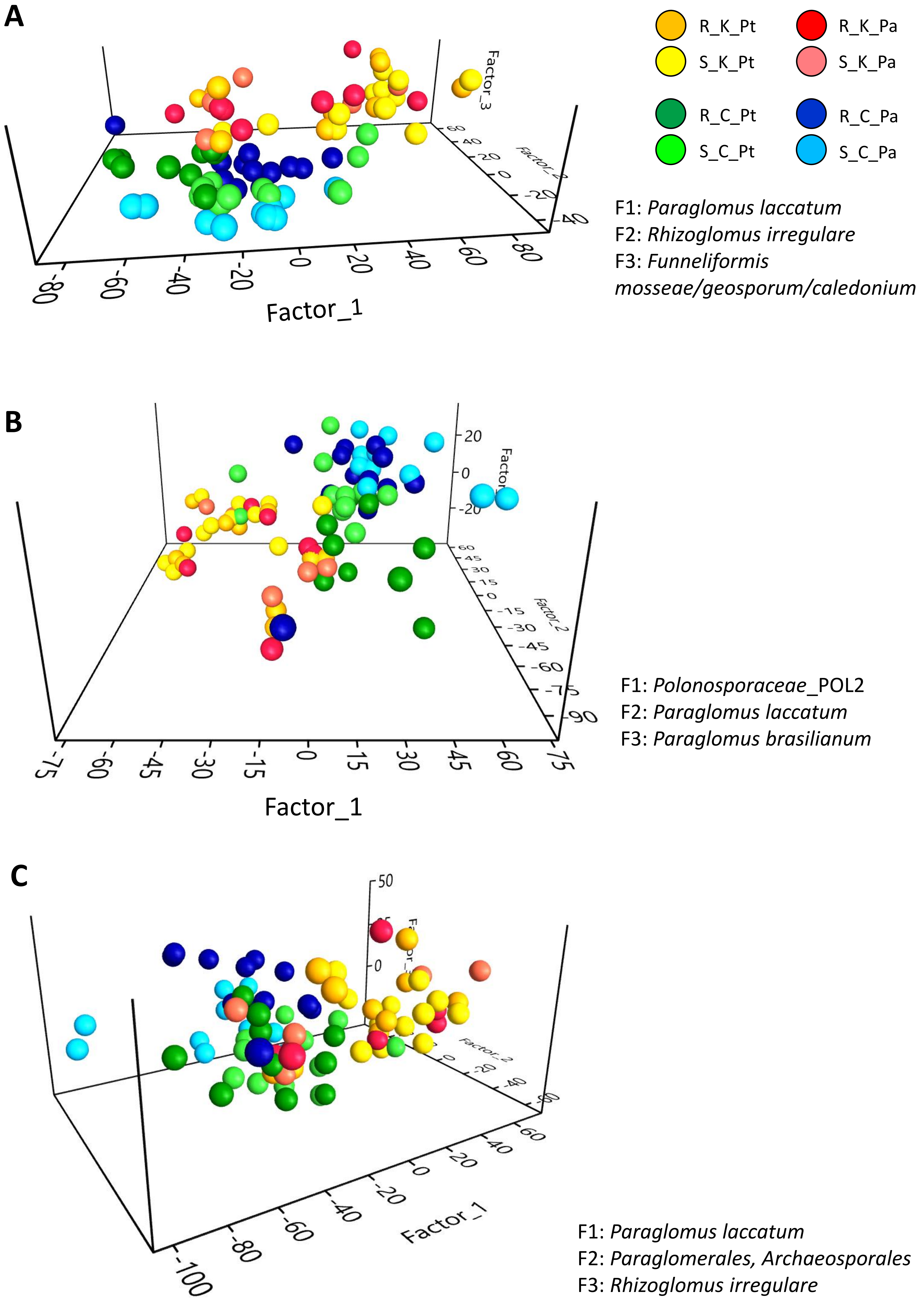

Figure S6. The ordination based on Placement-Factorization showing the distribution of root (R) and soil (S) AMF communities associated with *P. trivialis* (Pt) and *Ph. australis* (Pa) at the contaminated (K) and the uncontaminated site (C). Placement-Factorization indicate the relationship between changes in the abundance of clades in the phylogenetic tree and changes is soil-physicochemical features assessed by the Generalized linear model (GLM). Axes represent balances of the winning edges of the first three phylofactors (F1, F2 and F3). Placement-Factorization was performed considering the effects of moisture/pH H_2O (**A**), pH KCl (**B**) and SOM content (C).
