# Supplementary material for: Phenol and Polyaromatic Hydrocarbons Are Stronger Drivers Than Host Plant Species in Shaping the Arbuscular Mycorrhizal Fungal Component of the Mycorrhizosphere
Source: Int J Mol Sci. 2022 Oct 20;23(20):12585. doi: 10.3390/ijms232012585 (PMC9604154; doi:10.3390/ijms232012585)
Supplement: Supplementary file 1 [file ijms-23-12585-s001.zip › Figure S7.pdf]

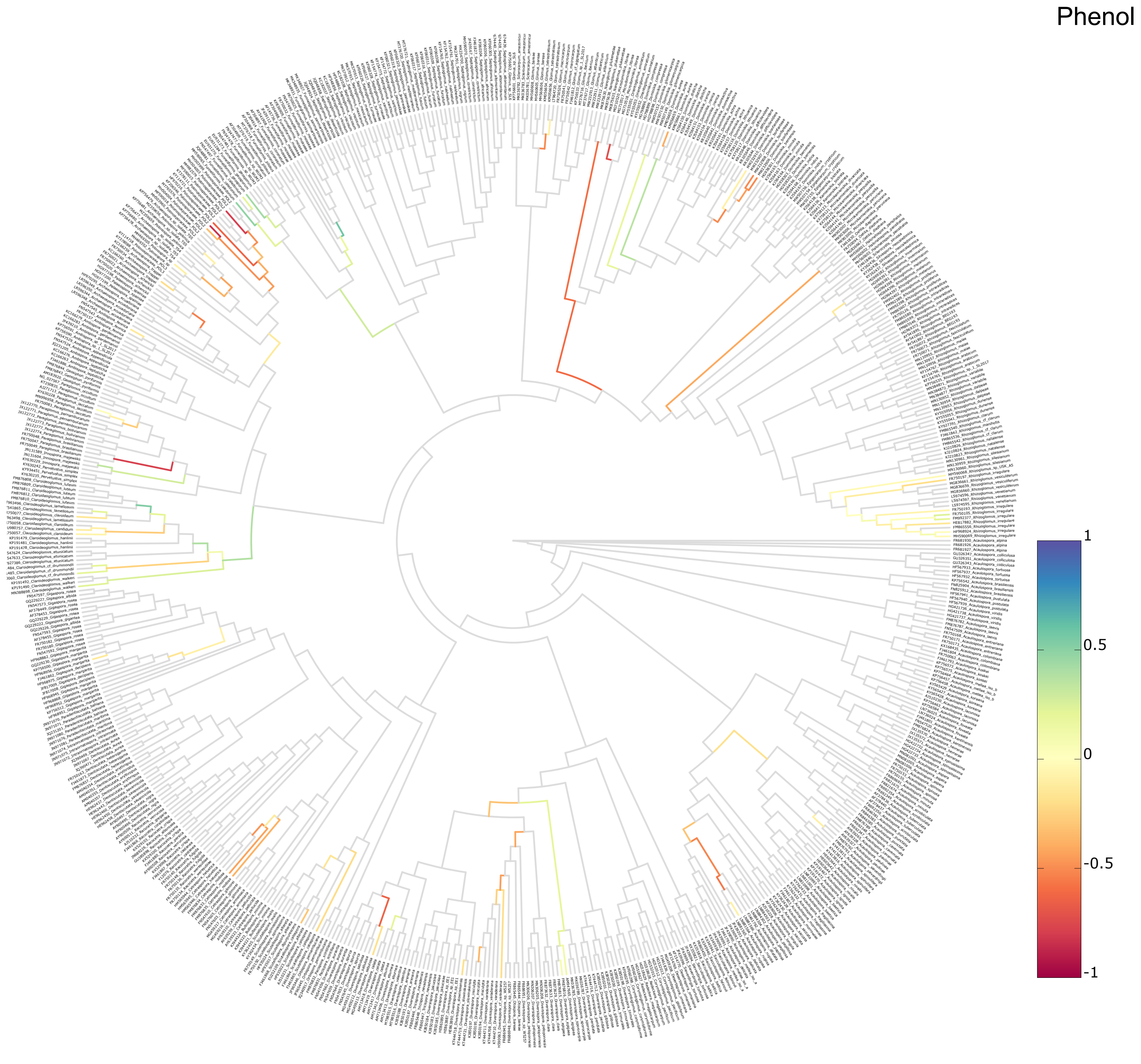
Phenol
1
0.5
0
-0.5
-1

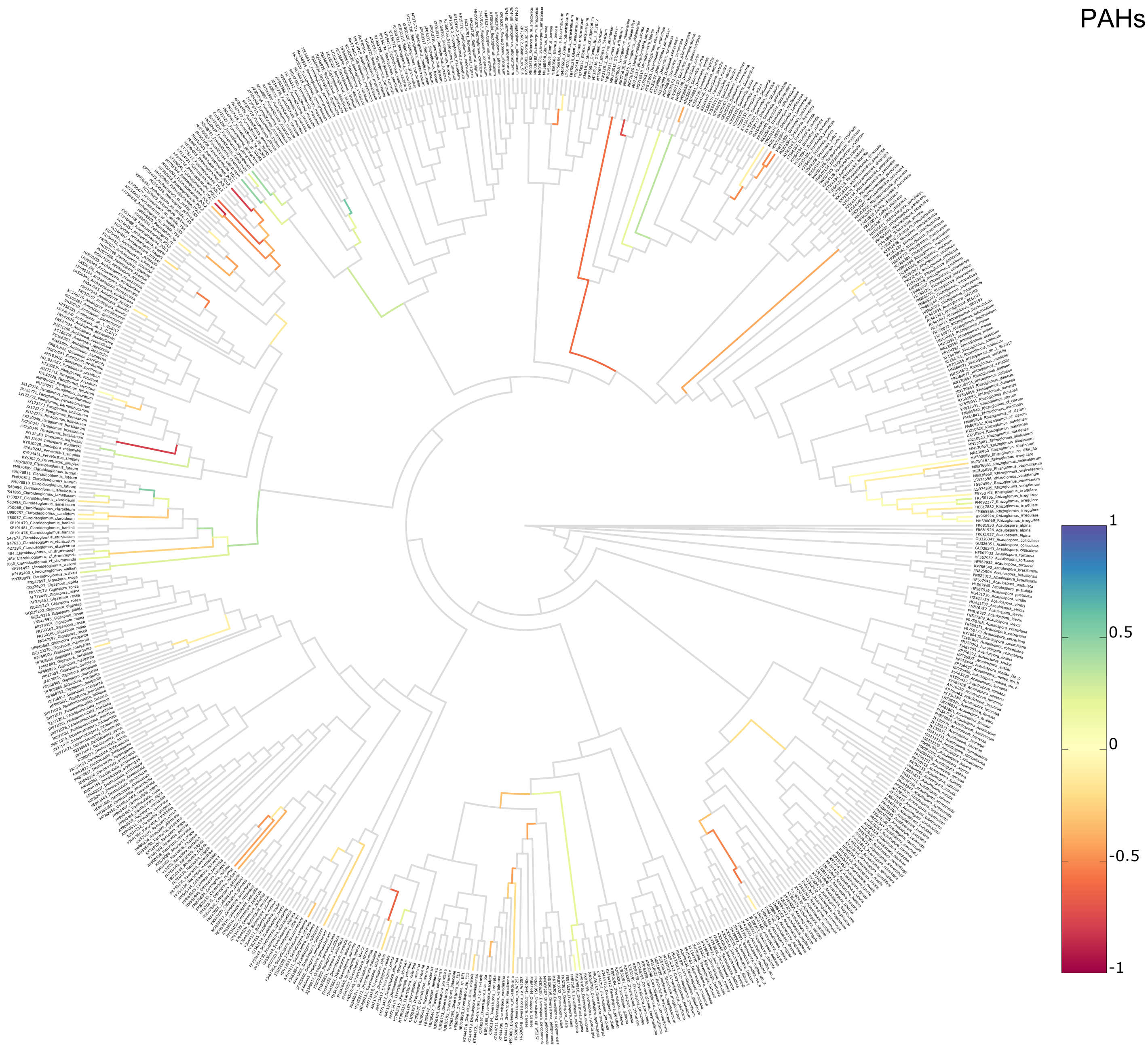
PAHs
1
0.5
0
-0.5
-1

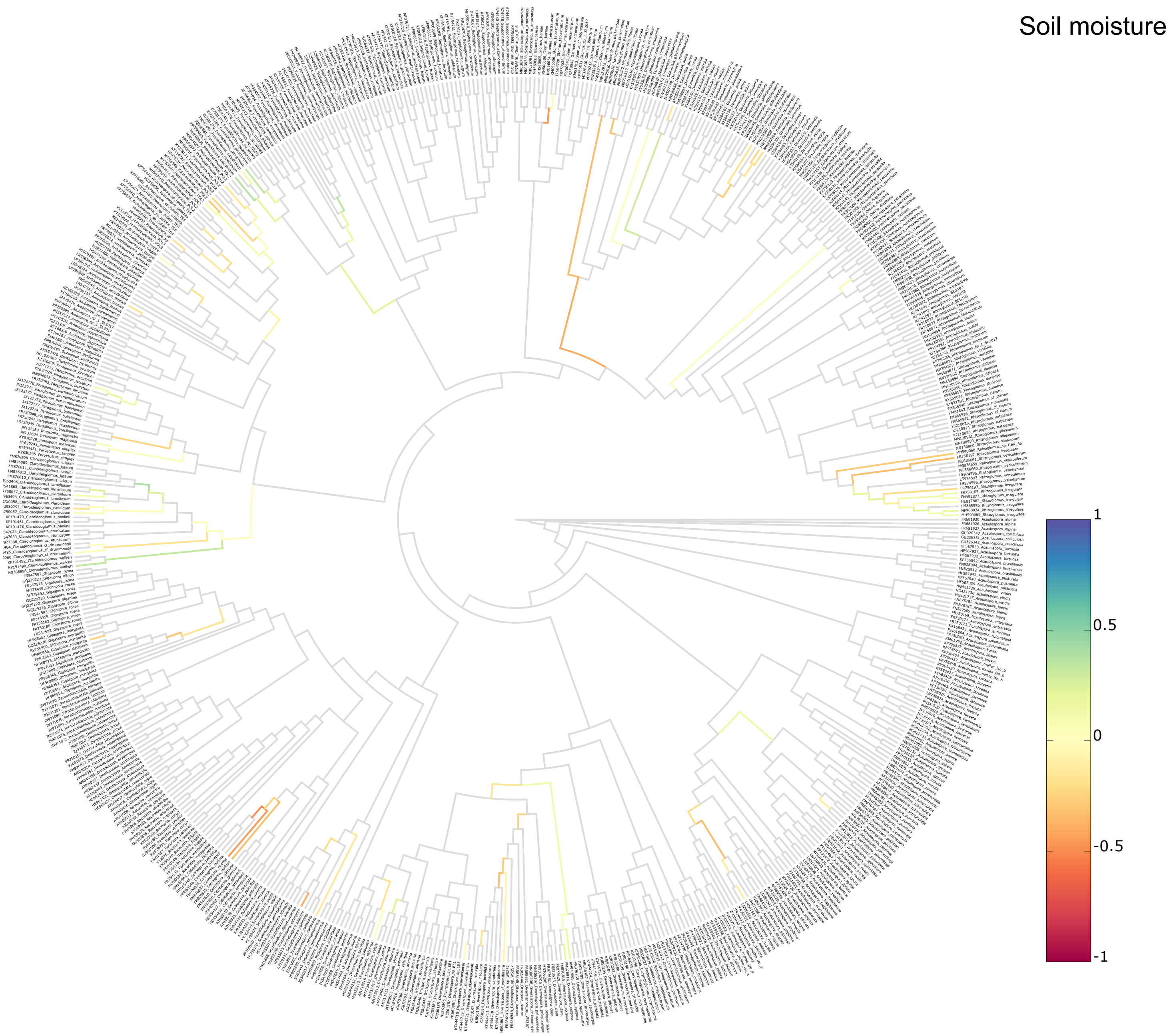
Soil moisture
1
0.5
0
-0.5
-1

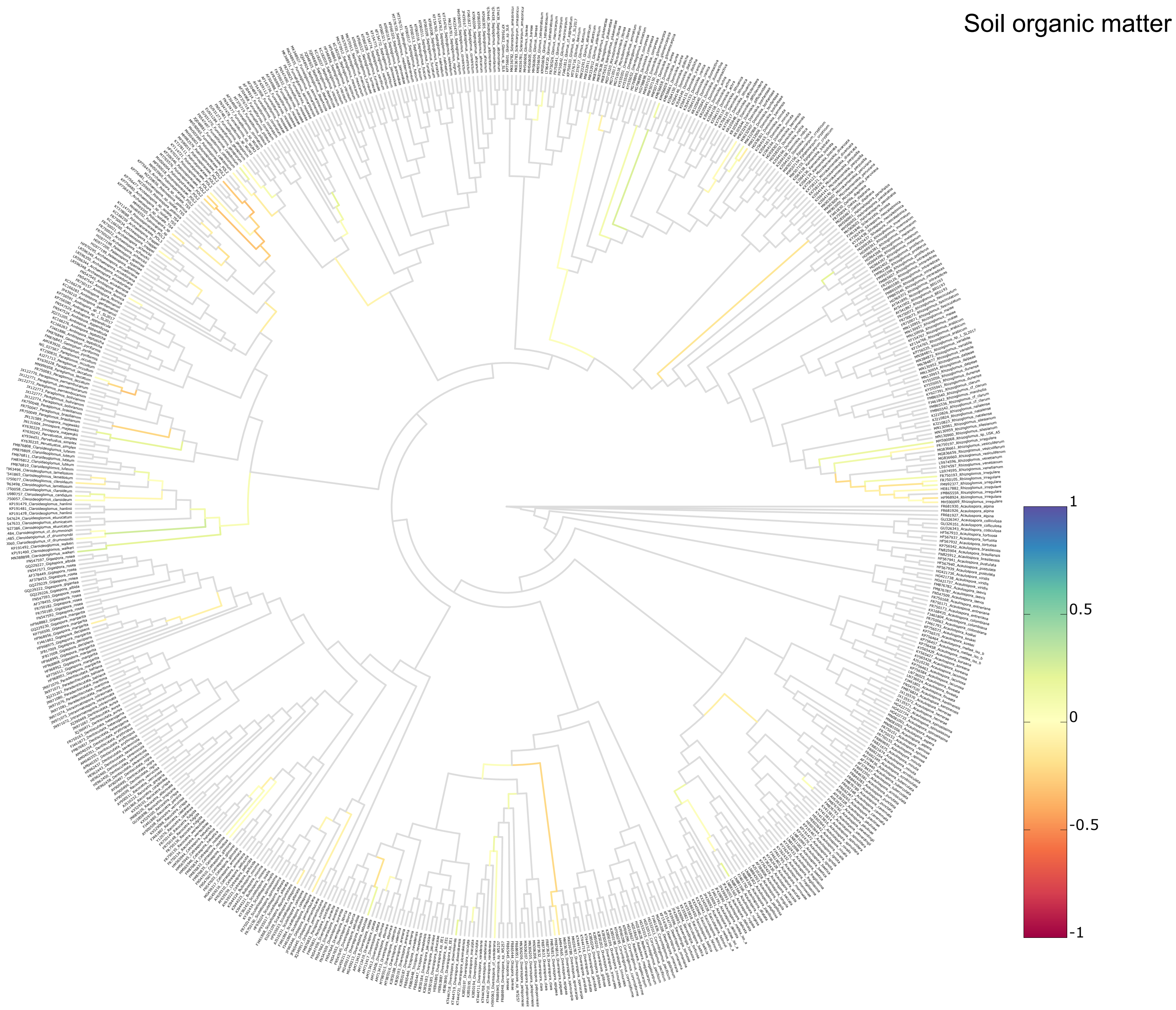
Soil organic matter
1
0.5
0
-0.5
-1

Total Nitrogen

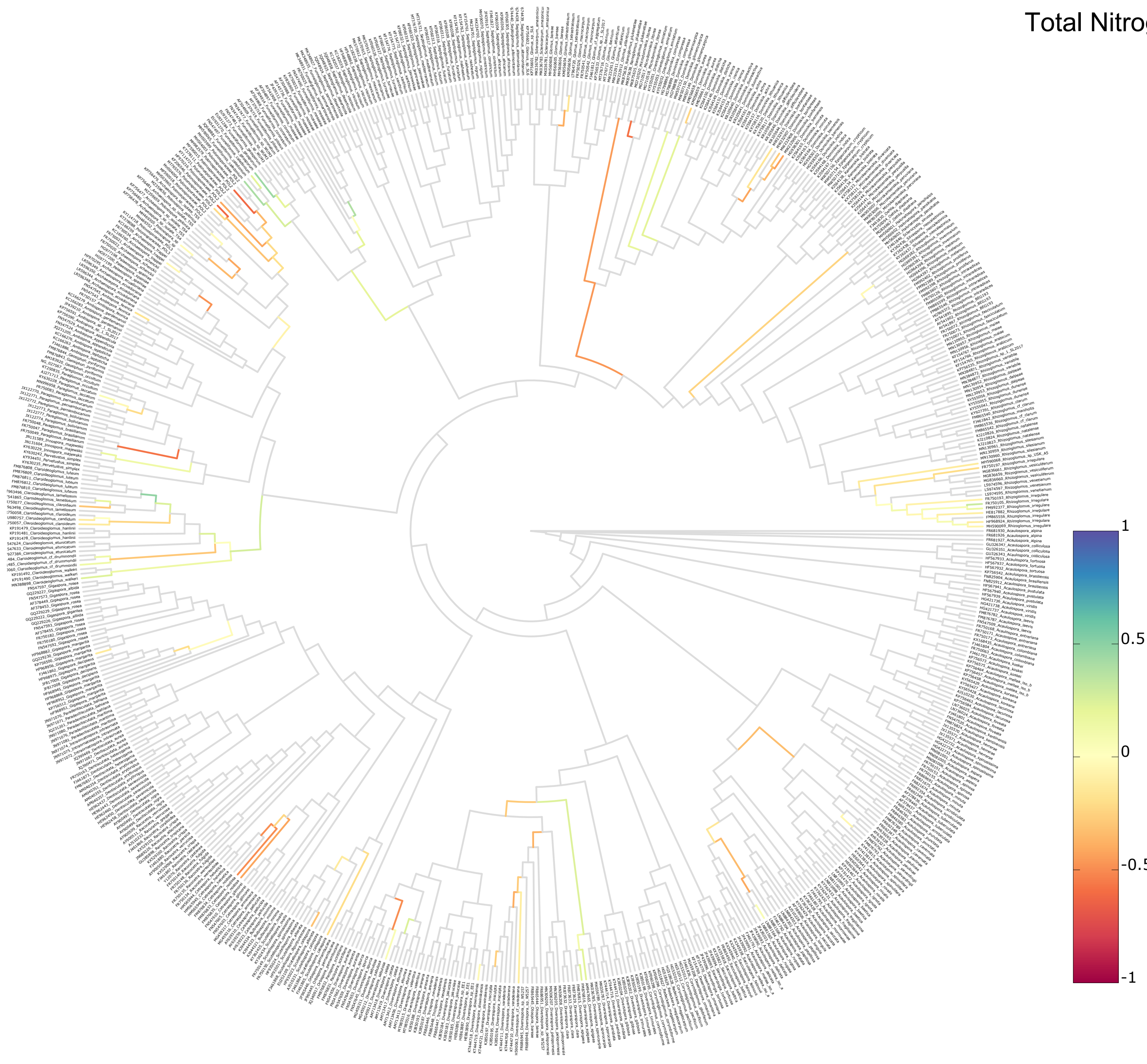

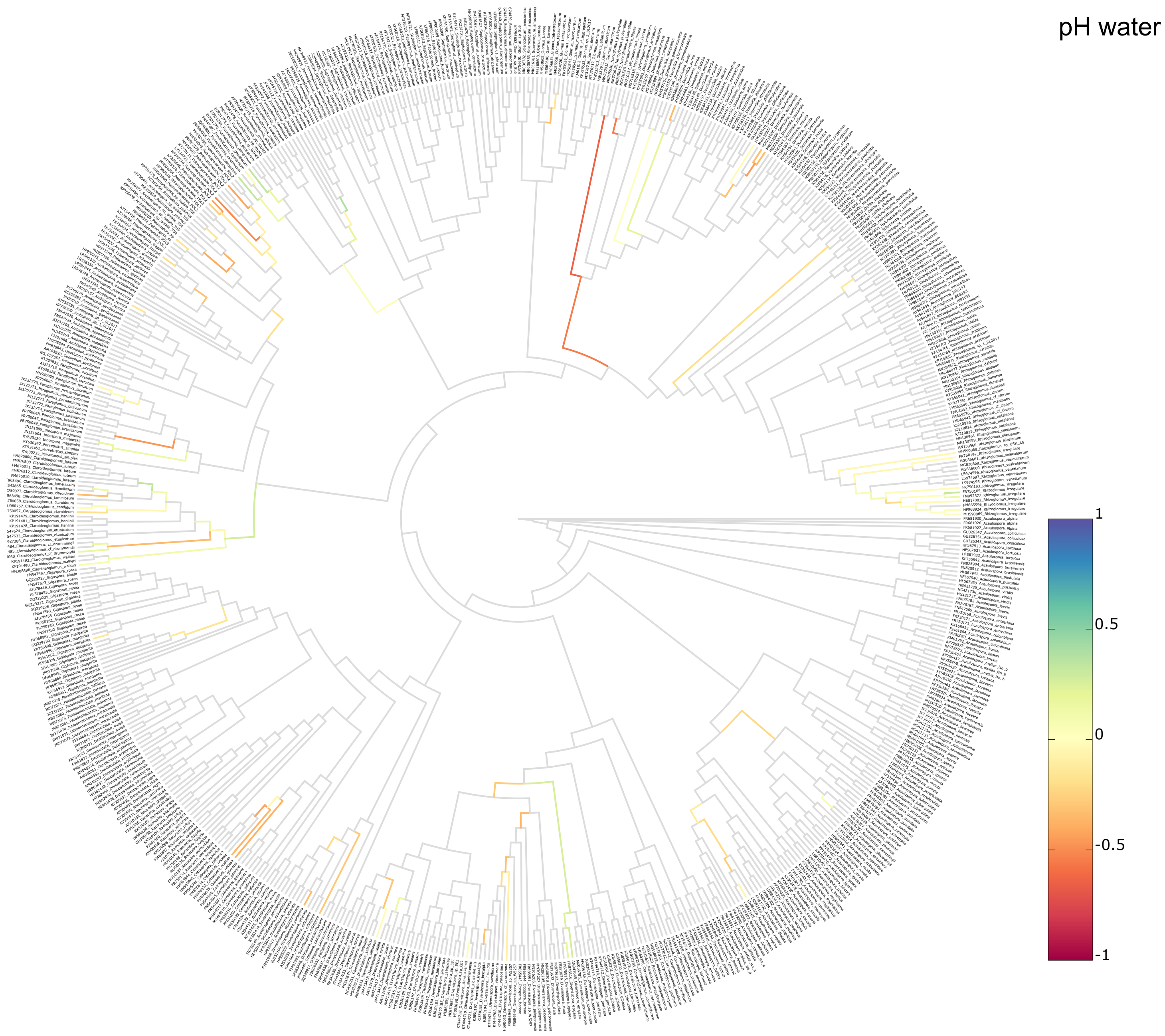
pH water
1
0.5
0
-0.5
-1

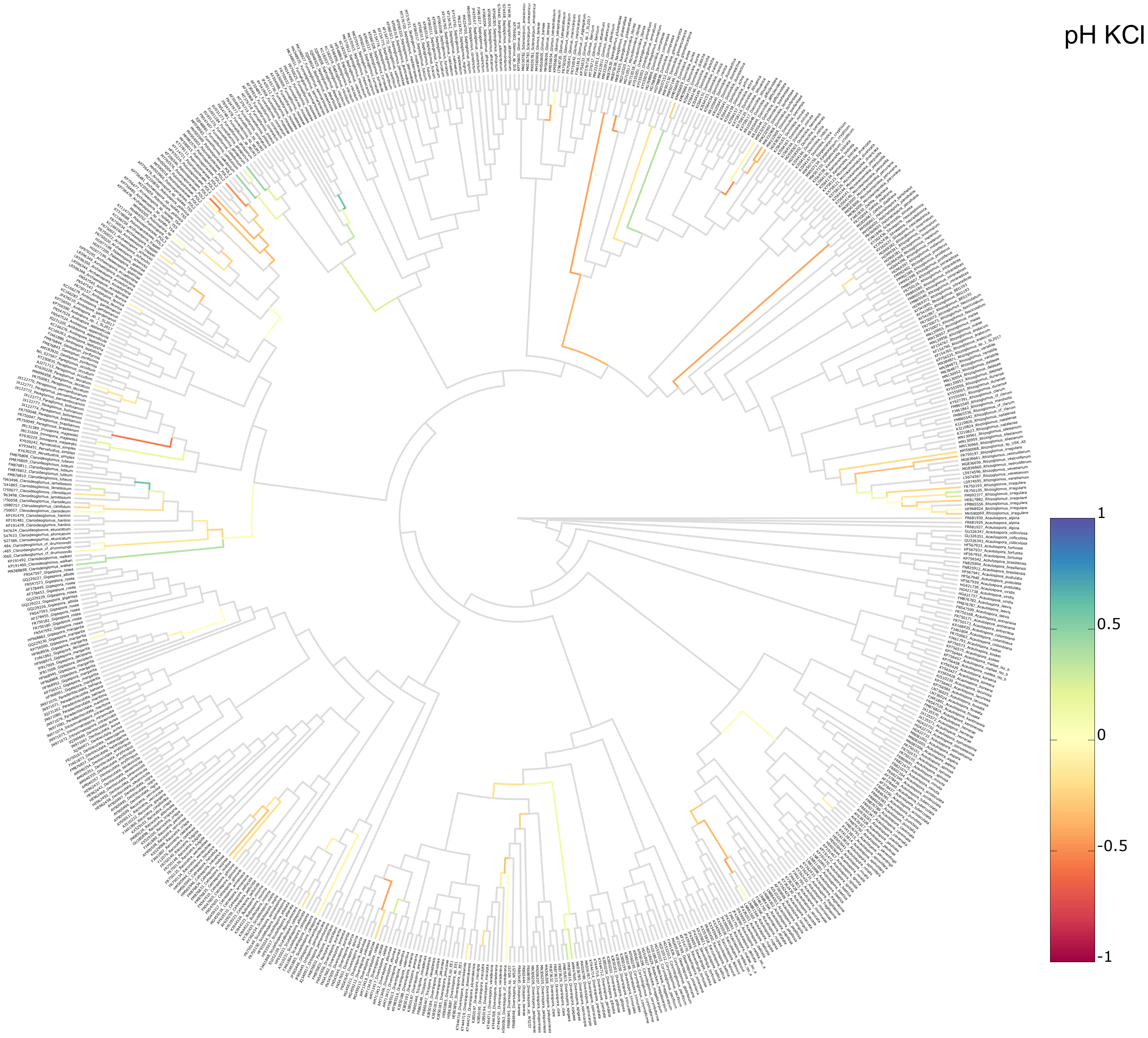

Figure S7. Trees visualizing Spearman's correlation coefficient between the studied soil physicochemical features (phenol, PAHs, moisture, soil organic matter, total nitrogen, pH water, pH KCl) and edge masses. Blue and red colours indicate positive and negative correlation of placements with the analyzed physicochemical features, respectively.
