# Supplementary material for: Phenol and Polyaromatic Hydrocarbons Are Stronger Drivers Than Host Plant Species in Shaping the Arbuscular Mycorrhizal Fungal Component of the Mycorrhizosphere
Source: Int J Mol Sci. 2022 Oct 20;23(20):12585. doi: 10.3390/ijms232012585 (PMC9604154; doi:10.3390/ijms232012585)
Supplement: Supplementary file 1 [file ijms-23-12585-s001.zip › Figure S9.pdf]

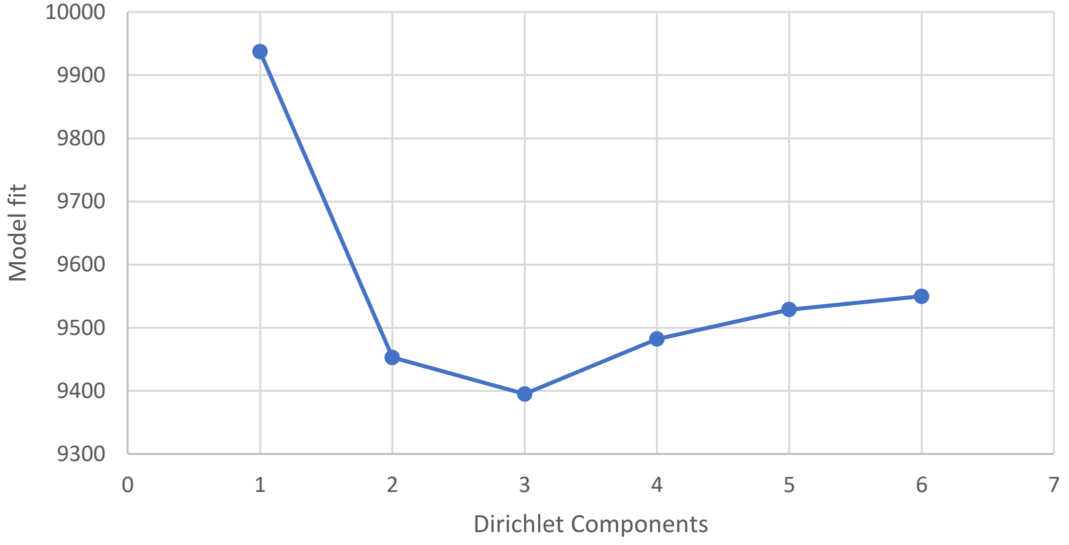


| Contaminated site | Control site/*P. trivialis* | Control site/*Ph.australis* |
|---|---|---|
| Partition 1 | Partition 2 | Partition 3 |
| R_C_Pt11 | R_C_Pa1 | R_C_Pa9 |
| R_K_Pa1 | R_C_Pa10 | R_C_Pt12 |
| R_K_Pa3 | R_C_Pa11 | R_C_Pt2 |
| R_K_Pa4 | R_C_Pa12 | R_C_Pt5 |
| R_K_Pa5 | R_C_Pa2 | R_C_Pt6 |
| R_K_Pa6 | R_C_Pa3 | R_C_Pt7 |
| R_K_Pa8 | R_C_Pa4 | R_C_Pt8 |
| R_K_Pt1 | R_C_Pa5 | R_C_Pt9 |
| R_K_Pt10 | R_C_Pa6 | S_C_Pa3 |
| R_K_Pt11 | R_C_Pa7 | S_C_Pa4 |
| R_K_Pt12 | R_C_Pa8 | S_C_Pa9 |
| R_K_Pt2 | R_C_Pt1 | S_C_Pt1 |
| R_K_Pt3 | R_C_Pt4 | S_C_Pt12 |
| R_K_Pt4 | S_C_Pa1 | S_C_Pt3 |
| R_K_Pt5 | S_C_Pa10 | S_C_Pt4 |
| R_K_Pt6 | S_C_Pa11 | S_C_Pt5 |
| R_K_Pt7 | S_C_Pa12 | S_C_Pt6 |
| R_K_Pt8 | S_C_Pa2 | S_C_Pt7 |
| R_K_Pt9 | S_C_Pa5 | S_C_Pt8 |
| S_K_Pa10 | S_C_Pa6 | S_C_Pt9 |
| S_K_Pa12 | S_C_Pa7 | |
| S_K_Pa3 | S_C_Pa8 | |
| S_K_Pa4 | S_C_Pt10 | |
| S_K_Pa6 | S_C_Pt2 | |
| S_K_Pa7 | | |
| S_K_Pa8 | | |
| S_K_Pt1 | | |
| S_K_Pt10 | | |
| S_K_Pt11 | | |
| S_K_Pt12 | | |
| S_K_Pt2 | | |
| S_K_Pt3 | | |
| S_K_Pt4 | | |
| S_K_Pt5 | | |
| S_K_Pt6 | | |
| S_K_Pt7 | | |
| S_K_Pt8 | | |
| S_K_Pt9 | | |

Figure S9. DMM model fit for the increasing number of Dirichlet mixture components (K) using the Laplace approximation to the negative log model evidence. The minimal Laplace value for K = 3 indicates the partitioning of samples to three significantly different clusters.
