# Supplementary material for: Phenol and Polyaromatic Hydrocarbons Are Stronger Drivers Than Host Plant Species in Shaping the Arbuscular Mycorrhizal Fungal Component of the Mycorrhizosphere
Source: Int J Mol Sci. 2022 Oct 20;23(20):12585. doi: 10.3390/ijms232012585 (PMC9604154; doi:10.3390/ijms232012585)
Supplement: Supplementary file 1 [file ijms-23-12585-s001.zip › Table S1.pdf]

Table S1. Physicochemical properties of soil samples.

Sample name	Phenol [mg kg^{-1} soil]	Naphthalene [mg kg-1 soil]	Phenanthrene [mg kg-1 soil]	Pyrene [mg kg-1 soil]	Fluorantene [mg kg-1 soil]	Dibenzo(a,h)anthracene [mg kg-1 soil]	Total PAHs [mg kg^{-1} soil]
Kalina PT 1	98.09	2.28	1.00	4.55	3.15	6.44	39.79
Kalina PT 2	251.83	2.81	1.90	5.58	3.98	6.49	51.34
Kalina PT 3	681.63	3.05	2.97	6.57	5.52	6.14	51.59
Kalina PT 4	736.16	2.43	1.74	5.11	3.62	8.19	53.23
Kalina PT 5	763.79	3.30	2.38	5.77	5.08	9.03	54.73
Kalina PT 6	939.00	2.39	3.18	4.90	8.50	7.99	64.89
Kalina PT 7	939.98	4.52	3.60	10.01	7.17	10.58	76.85
Kalina PT 8	1142.45	4.26	3.99	9.60	7.38	12.39	88.61
Kalina PT 9	1334.06	6.55	4.76	15.94	10.90	12.97	105.00
Kalina PT 10	1441.09	57.78	33.09	67.38	60.35	33.81	394.40
Kalina PT 11	2813.76	69.40	52.20	87.40	86.41	34.86	503.40
Kalina PT 12	1940.59	77.15	52.10	84.50	12.38	38.25	445.72
Kalina PA 1	100.01	2.54	0.87	5.03	4.72	5.80	27.74
Kalina PA 2	164.76	1.96	2.67	4.70	4.80	5.32	56.89
Kalina PA 3	734.87	2.69	2.63	6.93	6.03	6.15	49.20
Kalina PA 4	745.92	3.16	1.71	4.91	4.65	9.15	60.80
Kalina PA 5	764.10	2.82	1.96	5.20	5.97	8.54	61.51
Kalina PA 6	921.65	2.97	3.64	5.82	7.92	8.67	67.30
Kalina PA 7	940.34	4.21	3.26	8.87	7.23	14.98	78.52
Kalina PA 8	1045.86	4.75	4.48	10.20	9.13	14.52	79.35
Kalina PA 9	1328.32	6.84	3.98	17.85	12.10	14.98	123.56
Kalina PA 10	1569.62	47.29	45.72	62.59	54.36	28.54	438.02
Kalina PA 11	2653.95	78.24	42.87	83.81	89.31	30.69	538.45
Kalina PA 12	1769.36	82.18	57.40	97.65	25.80	29.51	472.87
Control PT 1	< 0.01	< 0.01	< 0.01	< 0.01	< 0.01	< 0.01	< 0.01
Control PT 2	< 0.01	< 0.01	< 0.01	< 0.01	< 0.01	< 0.01	< 0.01
Control PT 3	< 0.01	< 0.01	< 0.01	< 0.01	< 0.01	< 0.01	< 0.01
Control PT 4	< 0.01	< 0.01	< 0.01	< 0.01	< 0.01	< 0.01	< 0.01
Control PT 5	< 0.01	< 0.01	< 0.01	< 0.01	< 0.01	< 0.01	< 0.01
Control PT 6	< 0.01	< 0.01	< 0.01	< 0.01	< 0.01	< 0.01	< 0.01
Control PT 7	< 0.01	< 0.01	< 0.01	< 0.01	< 0.01	< 0.01	< 0.01
Control PT 8	< 0.01	< 0.01	< 0.01	< 0.01	< 0.01	< 0.01	< 0.01
Control PT 9	< 0.01	< 0.01	< 0.01	< 0.01	< 0.01	< 0.01	< 0.01
Control PT 10	< 0.01	< 0.01	< 0.01	< 0.01	< 0.01	< 0.01	< 0.01
Control PT 11	< 0.01	< 0.01	< 0.01	< 0.01	< 0.01	< 0.01	< 0.01
Control PT 12	< 0.01	< 0.01	< 0.01	< 0.01	< 0.01	< 0.01	< 0.01
Control PA 1	< 0.01	< 0.01	< 0.01	< 0.01	< 0.01	< 0.01	< 0.01
Control PA 2	< 0.01	< 0.01	< 0.01	< 0.01	< 0.01	< 0.01	< 0.01
Control PA 3	< 0.01	< 0.01	< 0.01	< 0.01	< 0.01	< 0.01	< 0.01
Control PA 4	< 0.01	< 0.01	< 0.01	< 0.01	< 0.01	< 0.01	< 0.01
Control PA 5	< 0.01	< 0.01	< 0.01	< 0.01	< 0.01	< 0.01	< 0.01
Control PA 6	< 0.01	< 0.01	< 0.01	< 0.01	< 0.01	< 0.01	< 0.01
Control PA 7	< 0.01	< 0.01	< 0.01	< 0.01	< 0.01	< 0.01	< 0.01
Control PA 8	< 0.01	< 0.01	< 0.01	< 0.01	< 0.01	< 0.01	< 0.01
Control PA 9	< 0.01	< 0.01	< 0.01	< 0.01	< 0.01	< 0.01	< 0.01
Control PA 10	< 0.01	< 0.01	< 0.01	< 0.01	< 0.01	< 0.01	< 0.01
Control PA 11	< 0.01	< 0.01	< 0.01	< 0.01	< 0.01	< 0.01	< 0.01
Control PA 12	< 0.01	< 0.01	< 0.01	< 0.01	< 0.01	< 0.01	< 0.01

Sample name	Soil moisture [%]	pH H_2O	pH KCl	Conductivity [µS cm^{-1}]	Soil organic matter [%]	Total N [g kg^{-1} soil]
Kalina PT 1	10	6.7	7.55	594	4.938	0.71
Kalina PT 2	7	7.23	7.74	720	10.773	0.65
Kalina PT 3	2	7.56	7.7	689	7.022	0.68
Kalina PT 4	8	7.28	7.76	916	5.068	0.7
Kalina PT 5	8	7.26	7.68	1492	1.766	0.7
Kalina PT 6	8	6.36	7.31	985	1.195	0.69
Kalina PT 7	6	6.98	7.75	1290	6.952	0.65
Kalina PT 8	11	6.73	7.72	1470	5.308	0.64
Kalina PT 9	5	7.01	6.61	361	4.694	0.68
Kalina PT 10	6	6.93	6.12	445	5.300	0.71
Kalina PT 11	7	7.15	7.5	387	4.219	0.63
Kalina PT 12	6	7.36	6.61	764	4.376	0.69
Kalina PA 1	3	7.12	7.22	1238	5.508	0.63
Kalina PA 2	7	7.02	7.78	225	4.987	0.62
Kalina PA 3	9	7.18	7.96	230	5.251	0.63
Kalina PA 4	8	7.66	7.83	819	3.146	0.7
Kalina PA 5	6	7.77	7.93	266	5.344	0.71
Kalina PA 6	8	6.98	6.54	831	3.365	0.65
Kalina PA 7	7	7.12	6.95	522	5.316	0.68
Kalina PA 8	5	7.3	6.59	267	3.728	0.68
Kalina PA 9	9	6.64	6.34	876	3.817	0.69
Kalina PA 10	7	7.22	7.58	921	2.907	0.71
Kalina PA 11	6	7.33	7.38	948	6.914	0.66
Kalina PA 12	6	7.21	7.26	1110	4.558	0.68
Control PT 1	4	6.64	6.53	95.9	5.416	0.6
Control PT 2	3	6.43	7.36	85.5	3.934	0.61
Control PT 3	2	6.6	6.84	154	3.231	0.65
Control PT 4	2	6.83	6.59	60.2	3.954	0.59
Control PT 5	5	6.16	6.7	166.5	4.742	0.59
Control PT 6	6	6.73	6.33	72.6	5.530	0.66
Control PT 7	4	5.93	6.98	85.6	5.077	0.61
Control PT 8	2	6.81	6.4	182	3.019	0.59
Control PT 9	3	6.84	6.7	351	4.008	0.59
Control PT 10	2	6.07	6.71	322	3.034	0.63
Control PT 11	5	6.07	6.59	60.4	5.235	0.69
Control PT 12	4	5.87	6.1	50	4.282	0.63
Control PA 1	3	6.78	6.87	35	10.779	0.65
Control PA 2	7	6.71	6.85	30	3.661	0.61
Control PA 3	6	7	6.43	48	5.576	0.64
Control PA 4	3	7.01	6.56	56.3	4.576	0.62
Control PA 5	3	6.93	6.57	59.9	2.302	0.61
Control PA 6	3	6.9	6.52	54.7	3.849	0.59
Control PA 7	8	7.06	6.6	53.6	1.525	0.6
Control PA 8	3	6.94	6.31	135.1	2.330	0.62
Control PA 9	11	6.78	6.88	52.1	2.266	0.59
Control PA 10	13	6.6	6.09	51.4	2.547	0.61
Control PA 11	12	6.93	6.71	102.1	2.191	0.6
Control PA 12	5	6.8	6.7	98.5	2.020	0.64
