# Supplementary material for: Phenol and Polyaromatic Hydrocarbons Are Stronger Drivers Than Host Plant Species in Shaping the Arbuscular Mycorrhizal Fungal Component of the Mycorrhizosphere
Source: Int J Mol Sci. 2022 Oct 20;23(20):12585. doi: 10.3390/ijms232012585 (PMC9604154; doi:10.3390/ijms232012585)
Supplement: Supplementary file 1 [file ijms-23-12585-s001.zip › Table S2.pdf]

Table S2. The results of Metastats, non-parametric T-test showing significant differences ($P < 0.05$) in species abundance between the AMF communities associated with: the site contaminated with phenol and PAHs and the uncontaminated site (Table A), different plant species at the contaminated site (Table B), and different plant species at the uncontaminated site (Table C).

Table A

Species	Contaminated site			Uncontaminated site			*P*-value
	Mean	Variance	Std. Error	Mean	Variance	Std. Error	
Paraglomus_laccatum	0.347	0.125	0.058	0.133	0.027	0.024	0.001
Polonosporaceae_POL2_sp1	0.002	0.000	0.002	0.183	0.036	0.028	0.001
Polonosporaceae_POL2_sp5	0.000	0.000	0.000	0.116	0.038	0.029	0.001
Paraglomus_brasilianum	0.002	0.000	0.002	0.116	0.008	0.013	0.001
Rhizoglomus_irregulare	0.159	0.061	0.040	0.070	0.016	0.019	0.051
Nanoglomus_plukenetiae	0.003	0.000	0.002	0.150	0.051	0.034	0.001
Oehlia_diaphana	0.020	0.006	0.013	0.039	0.011	0.016	0.402
Claroideoglomus_luteum	0.075	0.019	0.023	0.000	0.000	0.000	0.001
Funneliformis_caledonium	0.095	0.053	0.038	0.000	0.000	0.000	0.001
Funneliformis_mosseae	0.086	0.041	0.033	0.000	0.000	0.000	0.001
Polonosporaceae_POL2	0.008	0.003	0.008	0.033	0.006	0.011	0.100
Polonospora_polonica	0.000	0.000	0.000	0.022	0.003	0.008	0.001
Microdominikia_litorea	0.000	0.000	0.000	0.017	0.002	0.007	0.001
Rhizoglomus	0.070	0.032	0.030	0.045	0.015	0.018	0.498
Polonospora	0.000	0.000	0.000	0.008	0.000	0.003	0.002
Claroideoglomus_walkeri	0.043	0.027	0.027	0.000	0.000	0.000	0.002
Polonosporaceae_POL2_sp6	0.000	0.000	0.000	0.003	0.000	0.001	0.001
Diversispora_sabulosa	0.000	0.000	0.000	0.004	0.000	0.001	0.001
Dominikia_aurea	0.014	0.002	0.007	0.003	0.000	0.001	0.114
Claroideoglomus_sp4	0.004	0.000	0.002	0.003	0.000	0.001	0.524
Dominikia_bonfanteae	0.000	0.000	0.000	0.006	0.000	0.001	0.001
Glomus_tetrastratosum	0.030	0.025	0.026	0.004	0.000	0.001	0.525
Claroideoglomus_claroideum	0.007	0.000	0.003	0.004	0.000	0.001	0.319
Polonosporaceae_POL2_sp8	0.000	0.000	0.000	0.002	0.000	0.001	0.002
Acaulospora_rugosa	0.000	0.000	0.000	0.003	0.000	0.001	0.001
Claroideoglomus_sp6	0.006	0.000	0.003	0.001	0.000	0.000	0.097
Cetraspora	0.000	0.000	0.000	0.006	0.000	0.002	0.001
Acaulospora_sp2	0.000	0.000	0.000	0.002	0.000	0.001	0.001
Glomeraceae	0.000	0.000	0.000	0.004	0.000	0.001	0.001
Cetraspora_nodosa	0.000	0.000	0.000	0.005	0.000	0.002	0.001
Archaeospora	0.000	0.000	0.000	0.002	0.000	0.000	0.001

Archaeospora_trappei	0.003	0.000	0.002	0.001	0.000	0.000	0.743
Diversisporaceae	0.000	0.000	0.000	0.002	0.000	0.001	0.019
Rhizoglomus_sp	0.004	0.000	0.002	0.007	0.000	0.003	0.491
Claroideoglomus_cf_drummondii	0.001	0.000	0.001	0.002	0.000	0.001	0.702
Polonosporaceae	0.000	0.000	0.000	0.002	0.000	0.002	0.397
Diversispora_epigaea	0.007	0.001	0.005	0.000	0.000	0.000	0.011
Funneliformis	0.001	0.000	0.000	0.000	0.000	0.000	0.001
Funneliformis_geosporum	0.007	0.001	0.004	0.000	0.000	0.000	0.001
Acaulospora_sp1	0.000	0.000	0.000	0.001	0.000	0.000	0.053
Polonosporaceae_POL2_sp7	0.000	0.000	0.000	0.000	0.000	0.000	0.001
Claroideoglomus_sp2	0.002	0.000	0.001	0.000	0.000	0.000	0.006
Diversisporaceae_sp1	0.000	0.000	0.000	0.000	0.000	0.000	0.001
Diversispora	0.000	0.000	0.000	0.000	0.000	0.000	0.001
Polonosporaceae_POL2_sp2	0.000	0.000	0.000	0.000	0.000	0.000	0.541
Scutellospora_calospora	0.000	0.000	0.000	0.001	0.000	0.000	0.001
Innospora_majewskii	0.000	0.000	0.000	0.000	0.000	0.000	0.022
Diversispora_cf_varaderana	0.000	0.000	0.000	0.000	0.000	0.000	0.948
Acaulospora_sp3	0.000	0.000	0.000	0.000	0.000	0.000	0.001
Rhizoglomus_sp2	0.000	0.000	0.000	0.000	0.000	0.000	0.647
Diversispora_insculpta	0.000	0.000	0.000	0.000	0.000	0.000	0.001
Diversispora_sp2	0.001	0.000	0.001	0.000	0.000	0.000	0.032
Dominikia_glomerocarpica	0.000	0.000	0.000	0.000	0.000	0.000	0.901
Diversispora_celata	0.001	0.000	0.000	0.000	0.000	0.000	0.028
Polonospora_sp	0.000	0.000	0.000	0.000	0.000	0.000	0.001
Claroideoglomus_sp1	0.000	0.000	0.000	0.000	0.000	0.000	0.140
Scutellospora	0.000	0.000	0.000	0.000	0.000	0.000	0.241
Diversispora_sp1	0.000	0.000	0.000	0.000	0.000	0.000	0.008
Polonosporaceae_POL2_sp3	0.000	0.000	0.000	0.000	0.000	0.000	0.001
Gigaspora_margarita	0.000	0.000	0.000	0.000	0.000	0.000	0.443
Claroideoglomus_candidum	0.000	0.000	0.000	0.000	0.000	0.000	0.003
Diversispora_slowinskensis	0.000	0.000	0.000	0.000	0.000	0.000	1.000
Polonosporaceae_POL2_sp4	0.000	0.000	0.000	0.000	0.000	0.000	1.000
Ambispora_fennica	0.000	0.000	0.000	0.000	0.000	0.000	1.000
Claroideoglomus_lamellosum	0.000	0.000	0.000	0.000	0.000	0.000	0.000
Gigaspora	0.000	0.000	0.000	0.000	0.000	0.000	1.000
Claroideoglomus_sp5	0.000	0.000	0.000	0.000	0.000	0.000	1.000
Gigaspora_sp3	0.000	0.000	0.000	0.000	0.000	0.000	1.000
Cetraspora_sp1	0.000	0.000	0.000	0.000	0.000	0.000	1.000
Acaulospora_sieverdingii	0.000	0.000	0.000	0.000	0.000	0.000	1.000
Rhizoglomus_invermaium	0.000	0.000	0.000	0.000	0.000	0.000	1.000

Table B

Species	Contaminated site			Uncontaminated site			*P*-value
	Mean	Variance	Std. Error	Mean	Variance	Std. Error	
Paraglomus_laccatum	0.294	0.160	0.111	0.375	0.110	0.068	0.529
Polonosporaceae_POL2_sp1	0.000	0.000	0.000	0.003	0.000	0.003	0.785
Polonosporaceae_POL2_sp5	0.000	0.000	0.000	0.000	0.000	0.000	1.000
Paraglomus_brasilianum	0.000	0.000	0.000	0.004	0.000	0.004	0.758
Rhizoglomus_irregulare	0.109	0.034	0.051	0.186	0.075	0.056	0.317
Nanoglomus_plukenetiae	0.005	0.000	0.005	0.002	0.000	0.001	0.852
Oehlia_diaphana	0.037	0.017	0.036	0.011	0.001	0.007	0.730
Claroideoglomus_luteum	0.047	0.007	0.023	0.090	0.026	0.033	0.324
Funneliformis_caledonium	0.189	0.092	0.084	0.044	0.027	0.034	0.153
Funneliformis_mosseae	0.149	0.096	0.086	0.052	0.011	0.021	0.319
Polonosporaceae_POL2	0.000	0.000	0.000	0.013	0.004	0.013	0.129
Polonospora_polonica	0.000	0.000	0.000	0.000	0.000	0.000	1.000
Microdominikia_litorea	0.000	0.000	0.000	0.000	0.000	0.000	1.000
Rhizoglomus	0.017	0.004	0.017	0.099	0.046	0.044	0.145
Polonospora	0.000	0.000	0.000	0.000	0.000	0.000	1.000
Claroideoglomus_walkeri	0.009	0.001	0.009	0.061	0.041	0.041	0.140
Polonosporaceae_POL2_sp6	0.000	0.000	0.000	0.000	0.000	0.000	1.000
Diversispora_sabulosa	0.000	0.000	0.000	0.000	0.000	0.000	1.000
Dominikia_aurea	0.014	0.001	0.010	0.014	0.002	0.009	0.987
Claroideoglomus_sp4	0.004	0.000	0.003	0.005	0.000	0.003	0.766
Dominikia_bonfanteae	0.000	0.000	0.000	0.000	0.000	0.000	0.926
Glomus_tetrastratosum	0.084	0.071	0.074	0.000	0.000	0.000	0.240
Claroideoglomus_claroideum	0.006	0.000	0.005	0.007	0.000	0.004	0.866
Polonosporaceae_POL2_sp8	0.000	0.000	0.000	0.000	0.000	0.000	1.000
Acaulospora_rugosa	0.000	0.000	0.000	0.000	0.000	0.000	1.000
Claroideoglomus_sp6	0.000	0.000	0.000	0.009	0.000	0.004	0.048
Cetraspora	0.000	0.000	0.000	0.000	0.000	0.000	1.000
Acaulospora_sp2	0.000	0.000	0.000	0.000	0.000	0.000	1.000
Glomeraceae	0.000	0.000	0.000	0.000	0.000	0.000	1.000
Cetraspora_nodosa	0.000	0.000	0.000	0.000	0.000	0.000	1.000
Archaeospora	0.000	0.000	0.000	0.000	0.000	0.000	1.000
Archaeospora_trappei	0.000	0.000	0.000	0.004	0.000	0.003	0.132
Diversisporaceae	0.000	0.000	0.000	0.000	0.000	0.000	0.177
Rhizoglomus_sp	0.000	0.000	0.000	0.007	0.000	0.003	0.002
Claroideoglomus_cf_drummondii	0.000	0.000	0.000	0.002	0.000	0.001	0.013
Polonosporaceae	0.000	0.000	0.000	0.000	0.000	0.000	1.000

Diversispora_epigaea	0.018	0.002	0.014	0.001	0.000	0.000	0.189
Funneliformis	0.000	0.000	0.000	0.001	0.000	0.001	0.483
Funneliformis_geosporum	0.012	0.001	0.010	0.005	0.000	0.003	0.526
Acaulospora_sp1	0.000	0.000	0.000	0.000	0.000	0.000	1.000
Polonosporaceae_POL2_sp7	0.000	0.000	0.000	0.000	0.000	0.000	1.000
Claroideoglomus_sp2	0.000	0.000	0.000	0.003	0.000	0.002	0.022
Diversisporaceae_sp1	0.000	0.000	0.000	0.000	0.000	0.000	1.000
Diversispora	0.000	0.000	0.000	0.000	0.000	0.000	1.000
Polonosporaceae_POL2_sp2	0.000	0.000	0.000	0.000	0.000	0.000	0.424
Scutellospora_calospora	0.000	0.000	0.000	0.000	0.000	0.000	1.000
Innospora_majewskii	0.000	0.000	0.000	0.001	0.000	0.001	0.303
Diversispora_cf_varaderana	0.001	0.000	0.001	0.000	0.000	0.000	0.497
Acaulospora_sp3	0.000	0.000	0.000	0.000	0.000	0.000	1.000
Rhizoglomus_sp2	0.000	0.000	0.000	0.000	0.000	0.000	0.330
Diversispora_insculpta	0.000	0.000	0.000	0.000	0.000	0.000	1.000
Diversispora_sp2	0.003	0.000	0.003	0.000	0.000	0.000	0.769
Dominikia_glomerocarpica	0.000	0.000	0.000	0.001	0.000	0.001	0.001
Diversispora_celata	0.000	0.000	0.000	0.001	0.000	0.001	0.014
Polonospora_sp	0.000	0.000	0.000	0.000	0.000	0.000	1.000
Claroideoglomus_sp1	0.001	0.000	0.001	0.000	0.000	0.000	0.765
Scutellospora	0.000	0.000	0.000	0.000	0.000	0.000	1.000
Diversispora_sp1	0.000	0.000	0.000	0.000	0.000	0.000	1.000
Polonosporaceae_POL2_sp3	0.000	0.000	0.000	0.000	0.000	0.000	1.000
Gigaspora_margarita	0.000	0.000	0.000	0.000	0.000	0.000	1.000
Claroideoglomus_candidum	0.000	0.000	0.000	0.000	0.000	0.000	1.000
Diversispora_slowinskensis	0.000	0.000	0.000	0.000	0.000	0.000	1.000
Polonosporaceae_POL2_sp4	0.000	0.000	0.000	0.000	0.000	0.000	1.000
Ambispora_fennica	0.000	0.000	0.000	0.000	0.000	0.000	1.000
Claroideoglomus_lamellosum	0.000	0.000	0.000	0.000	0.000	0.000	0.247
Gigaspora	0.000	0.000	0.000	0.000	0.000	0.000	1.000
Claroideoglomus_sp5	0.000	0.000	0.000	0.000	0.000	0.000	1.000
Gigaspora_sp3	0.000	0.000	0.000	0.000	0.000	0.000	1.000
Cetraspora_sp1	0.000	0.000	0.000	0.000	0.000	0.000	1.000
Acaulospora_sieverdingii	0.000	0.000	0.000	0.000	0.000	0.000	1.000
Rhizoglomus_invermaium	0.000	0.000	0.000	0.000	0.000	0.000	1.000

Table C

Species	Contaminated site			Uncontaminated site			*P*-value
	Mean	Variance	Std. Error	Mean	Variance	Std. Error	
Paraglomus_laccatum	0.187	0.025	0.032	0.073	0.022	0.032	0.016
Polonosporaceae_POL2_sp1	0.276	0.045	0.044	0.078	0.004	0.014	0.001
Polonosporaceae_POL2_sp5	0.193	0.058	0.049	0.028	0.002	0.009	0.003
Paraglomus_brasilianum	0.123	0.005	0.015	0.107	0.011	0.023	0.556
Rhizoglomus_irregulare	0.042	0.013	0.024	0.103	0.018	0.030	0.107
Nanoglomus_plukenetiae	0.029	0.003	0.012	0.289	0.070	0.058	0.001
Oehlia_diaphana	0.071	0.019	0.028	0.001	0.000	0.000	0.001
Claroideoglomus_luteum	0.000	0.000	0.000	0.000	0.000	0.000	0.326
Funneliformis_caledonium	0.000	0.000	0.000	0.000	0.000	0.000	1.000
Funneliformis_mosseae	0.000	0.000	0.000	0.000	0.000	0.000	1.000
Polonosporaceae_POL2	0.011	0.000	0.004	0.057	0.011	0.023	0.041
Polonospora_polonica	0.020	0.005	0.014	0.023	0.001	0.007	0.851
Microdominikia_litorea	0.002	0.000	0.001	0.034	0.004	0.013	0.003
Rhizoglomus	0.003	0.000	0.002	0.093	0.028	0.036	0.002
Polonospora	0.007	0.001	0.005	0.008	0.000	0.004	0.901
Claroideoglomus_walkeri	0.000	0.000	0.000	0.000	0.000	0.000	1.000
Polonosporaceae_POL2_sp6	0.004	0.000	0.001	0.002	0.000	0.001	0.300
Diversispora_sabulosa	0.004	0.000	0.001	0.004	0.000	0.003	0.915
Dominikia_aurea	0.000	0.000	0.000	0.005	0.000	0.002	0.034
Claroideoglomus_sp4	0.004	0.000	0.002	0.001	0.000	0.001	0.132
Dominikia_bonfanteae	0.002	0.000	0.001	0.010	0.000	0.003	0.001
Glomus_tetrastratosum	0.001	0.000	0.000	0.008	0.000	0.002	0.004
Claroideoglomus_claroideum	0.001	0.000	0.001	0.006	0.000	0.002	0.012
Polonosporaceae_POL2_sp8	0.004	0.000	0.002	0.000	0.000	0.000	0.040
Acaulospora_rugosa	0.004	0.000	0.002	0.001	0.000	0.001	0.650
Claroideoglomus_sp6	0.000	0.000	0.000	0.001	0.000	0.001	0.164
Cetraspora	0.000	0.000	0.000	0.012	0.000	0.004	0.002
Acaulospora_sp2	0.003	0.000	0.001	0.002	0.000	0.001	0.531
Glomeraceae	0.001	0.000	0.000	0.007	0.000	0.002	0.001
Cetraspora_nodosa	0.000	0.000	0.000	0.011	0.000	0.004	0.001
Archaeospora	0.002	0.000	0.001	0.001	0.000	0.000	0.343
Archaeospora_trappei	0.001	0.000	0.000	0.001	0.000	0.001	0.899
Diversisporaceae	0.001	0.000	0.000	0.003	0.000	0.001	0.085
Rhizoglomus_sp	0.000	0.000	0.000	0.014	0.001	0.005	0.001
Claroideoglomus_cf_drummondii	0.000	0.000	0.000	0.004	0.000	0.001	0.002
Polonosporaceae	0.000	0.000	0.000	0.005	0.000	0.005	0.597

Diversispora_epigaea	0.000	0.000	0.000	0.000	0.000	0.000	1.000
Funneliformis	0.000	0.000	0.000	0.000	0.000	0.000	1.000
Funneliformis_geosporum	0.000	0.000	0.000	0.000	0.000	0.000	1.000
Acaulospora_sp1	0.000	0.000	0.000	0.001	0.000	0.001	0.307
Polonosporaceae_POL2_sp7	0.001	0.000	0.000	0.000	0.000	0.000	0.001
Claroideoglomus_sp2	0.000	0.000	0.000	0.000	0.000	0.000	1.000
Diversisporaceae_sp1	0.000	0.000	0.000	0.001	0.000	0.000	0.039
Diversispora	0.000	0.000	0.000	0.001	0.000	0.000	0.144
Polonosporaceae_POL2_sp2	0.000	0.000	0.000	0.000	0.000	0.000	0.002
Scutellospora_calospora	0.000	0.000	0.000	0.001	0.000	0.001	0.003
Innospora_majewskii	0.000	0.000	0.000	0.000	0.000	0.000	1.000
Diversispora_cf_varaderana	0.000	0.000	0.000	0.000	0.000	0.000	0.162
Acaulospora_sp3	0.000	0.000	0.000	0.000	0.000	0.000	0.385
Rhizoglomus_sp2	0.000	0.000	0.000	0.000	0.000	0.000	0.075
Diversispora_insculpta	0.000	0.000	0.000	0.000	0.000	0.000	0.114
Diversispora_sp2	0.000	0.000	0.000	0.000	0.000	0.000	1.000
Dominikia_glomerocarpica	0.000	0.000	0.000	0.000	0.000	0.000	1.000
Diversispora_celata	0.000	0.000	0.000	0.000	0.000	0.000	1.000
Polonospora_sp	0.000	0.000	0.000	0.000	0.000	0.000	0.525
Claroideoglomus_sp1	0.000	0.000	0.000	0.000	0.000	0.000	0.326
Scutellospora	0.000	0.000	0.000	0.000	0.000	0.000	0.054
Diversispora_sp1	0.000	0.000	0.000	0.001	0.000	0.001	0.001
Polonosporaceae_POL2_sp3	0.000	0.000	0.000	0.000	0.000	0.000	0.768
Gigaspora_margarita	0.000	0.000	0.000	0.000	0.000	0.000	0.122
Claroideoglomus_candidum	0.000	0.000	0.000	0.000	0.000	0.000	0.120
Diversispora_slowinskensis	0.000	0.000	0.000	0.000	0.000	0.000	0.077
Polonosporaceae_POL2_sp4	0.000	0.000	0.000	0.000	0.000	0.000	0.341
Ambispora_fennica	0.000	0.000	0.000	0.001	0.000	0.001	0.069
Claroideoglomus_lamellosum	0.000	0.000	0.000	0.000	0.000	0.000	1.000
Gigaspora	0.000	0.000	0.000	0.000	0.000	0.000	1.000
Claroideoglomus_sp5	0.000	0.000	0.000	0.000	0.000	0.000	0.000
Gigaspora_sp3	0.000	0.000	0.000	0.000	0.000	0.000	1.000
Cetraspora_sp1	0.000	0.000	0.000	0.000	0.000	0.000	1.000
Acaulospora_sieverdingii	0.000	0.000	0.000	0.000	0.000	0.000	0.000
Rhizoglomus_invermaium	0.000	0.000	0.000	0.000	0.000	0.000	1.000
