# Supplementary material for: Phenol and Polyaromatic Hydrocarbons Are Stronger Drivers Than Host Plant Species in Shaping the Arbuscular Mycorrhizal Fungal Component of the Mycorrhizosphere
Source: Int J Mol Sci. 2022 Oct 20;23(20):12585. doi: 10.3390/ijms232012585 (PMC9604154; doi:10.3390/ijms232012585)
Supplement: Supplementary file 1 [file ijms-23-12585-s001.zip › Table S3.pdf]

Table S3. The percentage of relative species abundance in the estimate of the mean of the reference single Dirichlet component (P0), and the three Dirichlet mixture components (P1, P2, P3) fitted to the studied AMF communities. Species are ranked in order of their contribution to the total mean difference of 262,8 %, split 75,9%, 75,8% and 75,8% across the components. The cumulative fraction of this difference is given in the last column.

Species	P0, mean	P1, mean	P2, mean	P3, mean	Difference	Cumulative fraction
Nanoglomus_plukenatiae	4.21	2.33	1.88	24.6	24.6	0.11
Polonosporaceae_POL2_sp1	5.12	1.04	20.55	8.84	23.24	0.21
Paraglomus_laccatum	14.5	19.24	19	4.46	19.29	0.3
Paraglomus_brasilianum	4.61	0.84	14.37	7.84	16.78	0.37
Rhizoglomus_irregulare	9.33	15.06	3.78	5.1	15.51	0.44
Polonosporaceae_POL2_sp5	3.45	0.22	9.29	3.11	9.41	0.48
Funneliformis_mosseae	1.34	7.11	0.06	0.01	8.38	0.52
Claroideoglomus_luteum	1.33	6.99	0.06	0.01	8.25	0.55
Funneliformis_caledonium	0.96	5.06	0.01	0.01	6.01	0.58
Rhizoglomus	3.56	5.04	1.41	1.94	5.25	0.6
Microdominikia_litorea	2.33	0.02	1.62	3.46	4.15	0.62
Funneliformis_geosporum	0.73	3.33	0.01	0.01	4.04	0.64
Glomeraceae	1.65	0.02	0.58	2.99	4.04	0.66
Claroideoglomus_claroideum	2.39	3.65	0.46	2.19	3.39	0.67
Oehlia_diaphana	3.42	2.62	3.89	1.37	3.33	0.68
Polonosporaceae_POL2	2.29	0.43	1.58	2.94	3.23	0.7
Rhizoglomus_sp	1.62	2.77	0.46	0.87	3.06	0.71
Claroideoglomus_sp4	2.74	3.09	1.38	1.48	2.97	0.73
Polonosporaceae_POL2_sp6	1.18	0.02	1.8	0.23	2.73	0.74
Cetraspora	1.08	0.02	0.3	1.84	2.6	0.75
Dominikia_bonfanteae	2.48	1.06	1.53	2.54	2.44	0.76
Diversispora_sabulosa	1.89	0.02	1.77	1.47	2.41	0.77
Glomus_tetrastratosum	2.25	1.27	1.04	2.42	2.37	0.78
Diversispora_epigaea	0.46	1.79	0.01	0.08	2.16	0.79
Polonosporaceae_POL2_sp7	1.09	0.02	1.31	0.22	2.16	0.8
Acaulospora_rugosa	1.45	0.02	1.42	0.78	2.13	0.81
Dominikia_aurea	1.35	2.07	0.12	1.44	2.03	0.82
Claroideoglomus_walkeri	0.36	1.66	0.01	0.01	2.02	0.83
Archaeospora	1.45	0.22	1.01	1.14	1.99	0.84
Cetraspora_nodosa	0.97	0.02	0.37	1.37	1.95	0.84
Diversispora	0.93	0.02	0.25	1.27	1.93	0.85
Acaulospora_sp2	1.25	0.02	1.1	0.76	1.88	0.86
Polonosporaceae_POL2_sp2	1.13	0.22	1.22	0.29	1.85	0.87

Diversisporaceae	1.54	0.64	0.77	1.39	1.81	0.88
Archaeospora_trappei	1.45	1.76	1.11	0.31	1.79	0.88
Polonospora_polonica	0.95	0.21	0.38	1.43	1.78	0.89
Polonospora	0.75	0.02	0.24	1.2	1.69	0.9
Claroideoglomus_sp1	0.34	1.25	0.06	0.01	1.53	0.91
Diversispora_insculpta	0.65	0.02	0.12	0.9	1.41	0.91
Claroideoglomus_cf_drummondii	1.2	1.09	0.18	1.47	1.4	0.92
Polonosporaceae_POL2_sp3	0.74	0.02	0.81	0.15	1.39	0.93
Diversisporaceae_sp1	0.51	0.02	0.06	0.81	1.23	0.93
Scutellospora_calospora	0.55	0.02	0.12	0.75	1.17	0.94
Claroideoglomus_sp6	0.64	1.12	0.01	0.65	1.13	0.94
Claroideoglomus_sp2	0.2	0.88	0.01	0.01	1.06	0.95
Innospora_majewskii	0.2	0.86	0.01	0.01	1.04	0.95
Funneliformis	0.2	0.85	0.01	0.01	1.04	0.95
Diversispora_celata	0.2	0.84	0.01	0.01	1.03	0.96
Polonosporaceae_POL2_sp8	0.57	0.02	0.54	0.25	0.91	0.96
Polonosporaceae_POL2_sp4	0.54	0.02	0.48	0.22	0.9	0.97
Acaulospora_sp3	0.5	0.02	0.45	0.23	0.82	0.97
Diversispora_cf_varaderana	0.82	0.84	0.12	0.89	0.79	0.97
Diversispora_sp2	0.15	0.64	0.01	0.01	0.78	0.98
Claroideoglomus_lamellosum	0.15	0.63	0.01	0.01	0.76	0.98
Diversispora_sp1	0.35	0.02	0.06	0.47	0.73	0.98
Acaulospora_sp1	0.2	0.02	0.01	0.34	0.51	0.99
Claroideoglomus_candidum	0.3	0.22	0.01	0.38	0.45	0.99
Diversispora_slowinskensis	0.2	0.02	0.18	0.08	0.32	0.99
Dominikia_glomerocarpica	0.05	0.22	0.01	0.01	0.26	0.99
Rhizoglomus_sp2	0.1	0.22	0.01	0.08	0.24	0.99
Gigaspora	0.15	0.02	0.12	0.08	0.23	0.99
Ambispora_fennica	0.1	0.02	0.01	0.15	0.23	0.99
Rhizoglomus_invermaium	0.1	0.02	0.01	0.15	0.23	0.99
Cetraspora_sp1	0.1	0.02	0.01	0.15	0.23	1
Scutellospora	0.15	0.02	0.06	0.15	0.22	1
Polonosporaceae	0.1	0.02	0.13	0.01	0.2	1
Gigaspora_margarita	0.1	0.02	0.06	0.08	0.14	1
Acaulospora_sieverdingii	0.05	0.02	0.01	0.08	0.11	1
Polonospora_sp	0.05	0.02	0.06	0.01	0.09	1
Claroideoglomus_sp5	0.05	0.02	0.06	0.01	0.09	1
Gigaspora_sp3	0.05	0.02	0.06	0.01	0.09	1
